# Topological indices and patterns in iron telluride networks

**DOI:** 10.1038/s41598-024-65205-y

**Published:** 2024-06-21

**Authors:** Hong Yang, Muhammad Farhan Hanif, Muhammad Kamran Siddiqui, Muhammad Faisal Hanif, Hira Ahmed, Samuel Asefa Fufa

**Affiliations:** 1 School of Computer Science, Chengdu University, Chengdu, China; 2 Department of Mathematics and Statistics, The University of Lahore, Lahore Campus, Lahore, Pakistan; 3 Department of Mathematics, COMSATS University Islamabad, Lahore Campus, Lahore, Pakistan; 4 Department of Mathematics, Addis Ababa University, Addis Ababa, Ethiopia

**Keywords:** Topological indices, General randic index, Zagreb type indices, Forgotten index, Balaban index, Iron telluride network, Applied mathematics, Physical chemistry

## Abstract

This paper explores the complex interplay between topological indices and structural patterns in networks of iron telluride (*FeTe*). We want to analyses and characterize the distinct topological features of (*FeTe*) by utilizing an extensive set of topological indices. We investigate the relationship that these indicators have with the network’s physical characteristics by employing sophisticated statistical techniques and curve fitting models. Our results show important trends that contribute to our knowledge of the architecture of the (*FeTe*) network and shed light on its physiochemical properties. This study advances the area of material science by providing a solid foundation for using topological indices to predict and analyses the behavior of intricate network systems. More preciously, we study the topological indices of iron telluride networks, an artificial substance widely used with unique properties due to its crystal structure. We construct a series of topological indices for iron telluride networks with exact mathematical analysis and determine their distributions and correlations using statistical methods. Our results reveal significant patterns and trends in the network structure when the number of constituent atoms increases. These results shed new light on the fundamental factors that influence material behavior, thus offering a deeper understanding of the iron telluride network and may contribute to future research and engineering of these materials.

## Introduction

A graph is any graphical representation made up of lines and points. Graphs that correlate to the constitutional formulas of chemical compounds are familiar to chemists. All of the circles represent atoms, while the lines connecting the respective circles represent chemical bounds^1^. In graph theory, the connecting lines are referred to as edges, while the items denoted by tiny circles are known as vertices^2^. Two sets make up the structure known as graphs $G=(V,E)$ in mathematics: the vertex set *V* and the edge set *E*^3^. The total number of edge connect with a vertex $\zeta$ is called its degree and is denoted by $\Upsilon (\zeta )$.

Networks of chemical compounds’ structures provide a strong foundation for comprehending and evaluating the characteristics of intricate molecular systems^10^. These networks offer a distinctive viewpoint on the connections between molecular structures and their attributes as they are built using the structural characteristics of chemical compounds. Chemical investigations revealed a high correlation between molecular structure’s topologies and their physical behaviors, chemical properties, and biological traits, including drug toxicity and melting and boiling points. Chen et al.^4^ discuss the design of multi-phase dynamic chemical networks. Avanzini et al.^5^ discuss the circuit theory for chemical reaction networks. The vertex-degree-based (abbreviated *VDB*) topological indices are a unique class of topological indices that are determined by the degree of the vertices in a (molecular) graph^7^. General Randic index used in chemical engineering to understand the connections between molecular structure and prospective physico-chemical properties^6^. Shaker et al.^8^ discus the superlattice via fifth zagreb topological polynomial. Zhang et al.^9^ derived results for multiplicative Zagreb indices of molecular graphs. Siddiqui et al.^11^ computed topological indices for chemical graphs.

In chemistry and related domains, the Randi$\acute{c}$ index is a graph theory descriptor used to measure the molecular structure of chemical compounds. A molecular graph’s Randi$\acute{c}$ index is determined by adding up the reciprocal square root of the product of the degrees of vertice pairs joined by an edge. This index is frequently used in quantitative structure-activity relationship (*QSAR*) investigations to predict the biological activity of chemical compounds. It offers insight into the topological arrangement of atoms in a molecule.

Then Bollobas et al.^12^ and Amic et al.^13^ worked independently and defined the generalized Randi$\acute{c}$ index as:

1 $$\begin{aligned} R_{\alpha }(G)=\sum \limits _{\zeta \varpi \in E(FeTe_{2})}\big (\Upsilon (\zeta )\times \Upsilon (\varpi )\big )^{\alpha }, \text {where }\alpha = 1,\, -1,\, \frac{1}{2},\, -\frac{1}{2} \end{aligned}$$

It gives the connection network of atoms inside a molecular structure a numerical representation. By adding up the products of the bond orders between atom pairs in a molecule, the index is determined. This descriptor contributes to the quantification of the molecular structure’s complexity and aids in the prediction of a number of attributes, including physicochemical traits and biological activity. Estrada et al.^14,15^ gave a new idea of an index and named as atom bond connectivity index:

2 $$\begin{aligned} ABC(G)= & {} \sum \limits _{\zeta \varpi \in E(FeTe_{2})}\sqrt{\frac{\Upsilon (\zeta )+\Upsilon (\varpi )-2}{\Upsilon (\zeta )\times \Upsilon (\varpi )}}. \end{aligned}$$

Vukicevic et al.^16^, defined the geometric arithmetic index as follwos:

3 $$\begin{aligned} GA(G)= & {} \sum \limits _{\zeta \varpi \in E(FeTe_{2})}\frac{2\sqrt{\Upsilon (\zeta )\times \Upsilon (\varpi )}}{\Upsilon (\zeta )+\Upsilon (\varpi )}. \end{aligned}$$

In the subject of Graph Theory, Gutman^17,18^ has made significant contributions. In terms of indices, he constructed the first and second Zagreb indices theoretically in 1972, as indicated by the following equations:

4 $$\begin{aligned} M_{1}(G)= & {} \sum \limits _{\zeta \varpi \in E(FeTe_{2})}(\Upsilon (\zeta )+\Upsilon (\varpi )). \end{aligned}$$

5 $$\begin{aligned} M_{2}(G)= & {} \sum \limits _{\zeta \varpi \in E(FeTe_{2})}(\Upsilon (\zeta )\times \Upsilon (\varpi )). \end{aligned}$$

Shirdal et al.^19^ introduced the hyper Zagreb index in 2013 as:

6 $$\begin{aligned} HM(G)= & {} \sum \limits _{\zeta \varpi \in E(FeTe_{2})}(\Upsilon (\zeta )+\Upsilon (\varpi ))^{2}. \end{aligned}$$

The first and second multiple Zagreb indices were introduced in 2012 by Ghorbani and Azimi^20^, who took into consideration the indices of any graph in the form of a product.

7 $$\begin{aligned} PM_{1}(G)= & {} \prod _{\zeta \varpi \in E(FeTe_{2})}(\Upsilon (\zeta )+\Upsilon (\varpi )). \end{aligned}$$

8 $$\begin{aligned} PM_{2}(G)= & {} \prod _{\zeta \varpi \in E(FeTe_{2})}(\Upsilon (\zeta )\times \Upsilon (\varpi )). \end{aligned}$$

The forgotten topological index, which Furtula and Gutman^21^ introduced, is a new method for indices when they are raised to the power of two. It can be calculated as follows:

9 $$\begin{aligned} F(G)= & {} \sum \limits _{\zeta \varpi \in E(FeTe_{2})}(\Upsilon (\zeta )^{2}+\Upsilon (\varpi )^{2}). \end{aligned}$$

Vukicevic et al.^22^ defined the Augmented Zagreb index as:

10 $$\begin{aligned} AZI(G)= & {} \sum \limits _{\zeta \varpi \in E(FeTe_{2})}\left[ \frac{(\Upsilon (\zeta ) \times \Upsilon (\varpi ))}{\Upsilon (\zeta ) + \Upsilon (\varpi )-2}\right] ^{3}. \end{aligned}$$

Balaban^23,24^ defined the Balaban index for a graph *G* with order $\acute{s}$ and size $\acute{r}$ as:

11 $$\begin{aligned} J(G)= & {} \bigg (\frac{\acute{r}}{\acute{r}-\acute{s}+2}\bigg )\left[ \sum \limits _{\zeta \varpi \in E(FeTe_{2})}\frac{1}{\sqrt{\Upsilon (\zeta )\times \Upsilon (\varpi )}}\right] . \end{aligned}$$

Guo et al.^25^ introduced the modeling concept of genes using graph-based approach and topological indices. Liu et al.^26^ discuss the quality model of clouds using degree based indices. Zhang et al.^27^ discuss the entropy in green building projects. Zhang et al.^28^ computed topological indices for ceria Oxide. Furtula et al.^29^ explore the Augmented Zagreb index(*AZI*) and also examine a forgotten topological index^30^. Ghorbani et al.^31^ give a brief overview of multiple Zagreb indices. Manzoor et al.^32,33^ discuused the degree-based entropy measures for metal-organic superlattices and hyaluronic acid-curcumin conjugates. Rashid et al.^34^ discuused the eccentricity-Based topoligcal indcies measures for Hypernetworks. Imran et al.^35^ computed the entropy measures of dendrimers. Siddiqui et al.^36^ computed the entropy measures for crystal structures.

**Figure 1:**
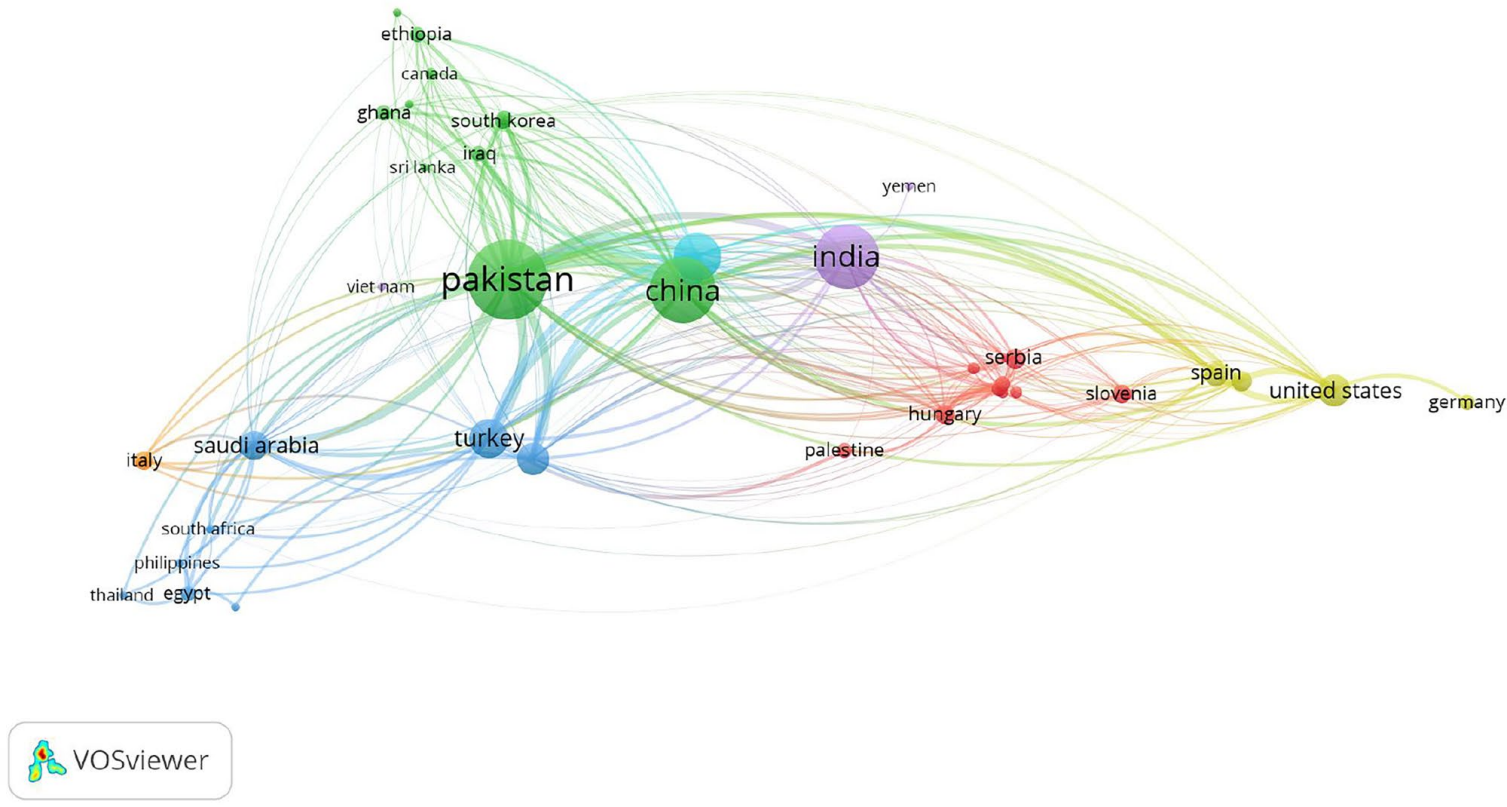
Bibliometric analysis: degree-based topological indices by various countries.

We have shown the bibliometric analysis of the degree-based topological indices by various nations throughout the world in Fig. 1.

**Figure 2:**
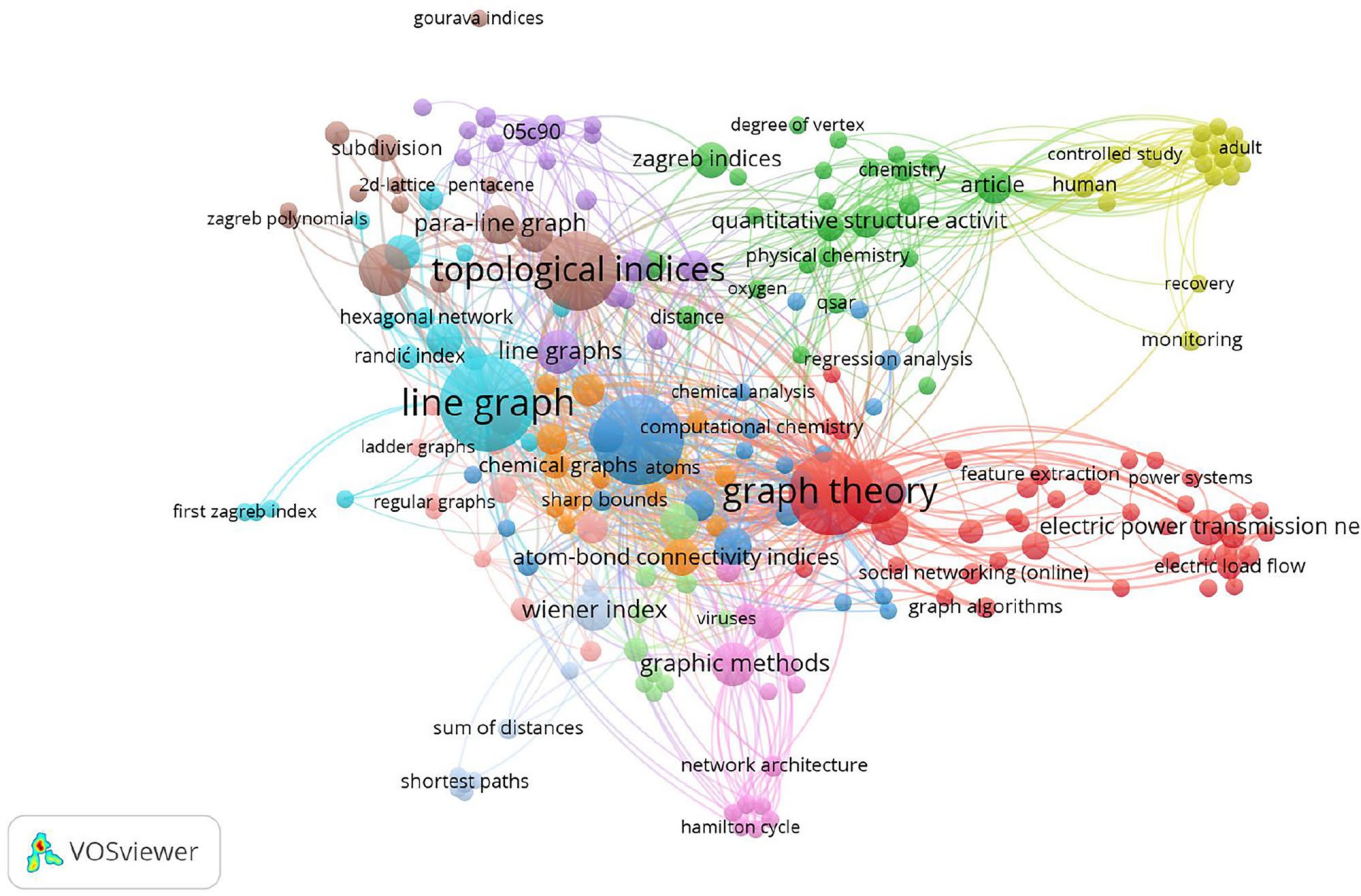
Bibliometric analysis: degree-based topological indices keywords.

Using the Scopus database (https://www.scopus.com/), this bibliometric analysis employs 1243 research publications on the application of topological indices in various domains. As seen below, we displayed the results of our bibliometric study of the topological Index terms in Fig. 2.

## Structure of iron telluride network $(FeTe_2)$

**Figure 3:**
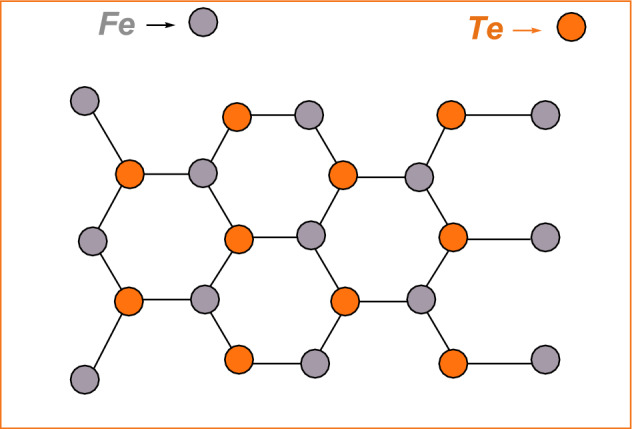
The $2\times 2$ sheet of Iron Telluride $(FeTe_2)$ network^38^.

Iron Telluride $(FeTe_2)$ is a compound that has gained significant attention in the field of materials science due to its unique properties. It belongs to the family of transition metal dichalcogenides (*TMDCs*), which are layered materials with a general formula of $MX_2$, where *M* is a transition metal and *X* is a chalcogen (sulfur, selenium, or tellurium). $FeTe_2$ has a layered crystal structure consisting of iron atoms sandwiched between two layers of tellurium atoms. Iron Telluride Network $(FeTe_2)$ is a two dimensional network shown in Fig. 3. The synthesis of Iron ditelluride can be achieved through various methods, including chemical vapor transport, chemical vapor deposition, and hydrothermal synthesis^37^. Since iron ditelluride exhibits distinct electrical and magnetic properties, it has garnered a lot of interest in the field of materials research. It displays a combined state of superconductivity and magnetism, making it a type-II superconductor. Because of this characteristic, it is a desirable material for a number of uses, such as energy storage, spintronics, and quantum computing^38^.

The Iron Telluride $(FeTe_2)$ formulas are computed by initially using unit cell is shown in Fig. 3. In order to compute the vertices, we now use Matlab software to generalize these formulas for vertices together with Table 1. The edge partition of iron telluride $(FeTe_2)$ will be taken as follows in order to determine its topological indices: The edge partition of iron telluride $(FeTe_2)$ with (*m*, *n*) is greater or equal to 1 is displayed in Table 2. The edge set is divided into five sets, let’s say $E_1$, $E_2$, $E_3$, $E_4$, and $E_5$, according to the degree of each edge’s end vertices. The order and size of $FeTe_{2}$ is $4mn+2m+n+1$ and $6mn+m$, respectively.

**Table 1:** Vertex Partition of $FeTe_{2}$.

$(\Upsilon (\zeta ))$	Total vertices	Set of vertices
1	$n+3$	$V_{1}$
2	$4m+n-3$	$V_{2}$
3	$4mn-2m-n+1$	$V_{3}$

**Table 2:** Edge Partition of $FeTe_{2}$.

$(\Upsilon (\zeta ),\, \Upsilon (\varpi ))$	Frequency	Set of edges
(1,2)	2	$E_{1}$
(1,3)	$n+1$	$E_{2}$
(2,2)	$2m-2$	$E_{3}$
(2,3)	$4m+2n-4$	$E_{4}$
(3,3)	$6mn-5m-3n+3$	$E_{5}$

## Results for iron telluride network $(FeTe_2)$

The derivation for General Randic index is:

$$\begin{aligned}{} & {} R_\alpha (G)=\sum \limits _{\zeta \varpi \in E(FeTe_{2})}(\Upsilon (\zeta )\times \Upsilon (\varpi ))^\alpha ;\, \, \, \ \alpha = 1,\, -1,\, \frac{1}{2},\, \frac{-1}{2}\\{} & {} {\textbf {For}}\,{\alpha = 1;}\\{} & {} R_1(FeTe_{2})=\sum \limits _{i=1}^{5}\sum \limits _{\zeta \varpi \in E_{i}(FeTe_{2})}(\Upsilon (\zeta ) \times \Upsilon (\varpi ))^{1}\\{} & {} \quad =(1\times 2)(2)+(1\times 3)(n+1)+(2\times 2)(2m-2)\\{} & {} \qquad +(2\times 3)(4m+2n-4)+(3\times 3)(6mn-5m-3n+3)\\{} & {} R_1(FeTe_{2})= 54mn - 13m - 12n + 2.\\{} & {} {\textbf {For}}\, {\alpha = -1;}\\{} & {} R_{-1}(FeTe_{2})=\sum \limits _{i=1}^{5}\sum \limits _{\zeta \varpi \in E_{i}(FeTe_{2})}(\Upsilon (\zeta )\times \Upsilon (\varpi ))^{-1}\\{} & {} \quad =\frac{1}{(1\times 2)}(2)+\frac{1}{(1\times 3)}(n+1)+\frac{1}{(2\times 2)}(2m-2)\\{} & {} \qquad +\frac{1}{(2\times 3)}(4m+2n-4)+\frac{1}{(3\times 3)}(6mn-5m-3n+3)\\{} & {} R_{-1}(FeTe_{2})= 0.6667mn + 0.6111m + 0.3333n + 0.5.\\{} & {} {\textbf {For}}\, {\alpha =\frac{1}{2};}\\{} & {} R_{\frac{1}{2}}(FeTe_{2})=\sum \limits _{i=1}^{5}\sum \limits _{\zeta \varpi \in E_{i}(FeTe_{2})}(\Upsilon (\zeta )\times \Upsilon (\varpi ))^{\frac{1}{2}}\\{} & {} \quad =\sqrt{(1\times 2)}(2)+\sqrt{1\times 3}(n+1)+\sqrt{(2\times 2)}(2m-2)\\{} & {} \qquad +\sqrt{(2\times 3)}(4m+2n-4)+\sqrt{(3\times 3)}(6mn-5m-3n+3)\\{} & {} R_{\frac{1}{2}}(FeTe_{2})=18mn - 1.20204m - 2.36897n - 0.237481.\\{} & {} {\textbf {For}}\, {\alpha = -\frac{1}{2};}\\{} & {} R_{-\frac{1}{2}}(FeTe_{2})=\sum \limits _{i=1}^{5}\sum \limits _{\zeta \varpi \in E_{i}(FeTe_{2})}\frac{1}{\sqrt{(\Upsilon (\zeta )\times \Upsilon (\varpi ))}}\\{} & {} \quad =\frac{1}{\sqrt{(1 \times 2)}}(2)+\frac{1}{\sqrt{1 \times 3}}(n+1)+\frac{1}{\sqrt{(2 \times 2)}}(2m-2)\\{} & {} \qquad +\frac{1}{\sqrt{(2 \times 3)}}(4m+2n-4)+\frac{1}{\sqrt{(3 \times 3)}}(6mn-5m-3n+3)\\{} & {} R_{-\frac{1}{2}}(FeTe_{2})=2mn + 0.966326m + 0.393847n + 0.358571. \end{aligned}$$

**Figure 4:**
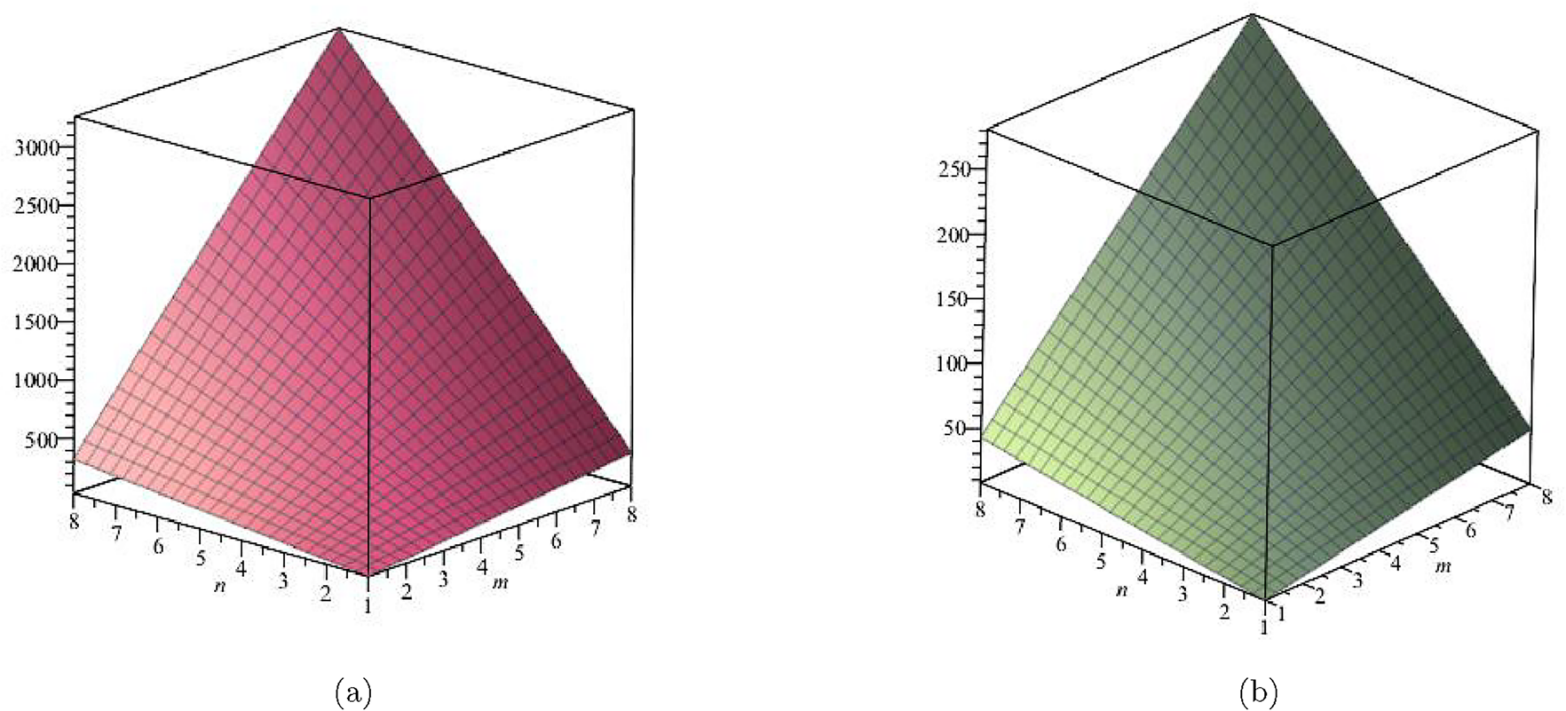
(**a**) Graph for ${R_{1}(FeTe_{2})}$,  (**b**) Graph for ${R_{-1}(FeTe_{2})}$.

**Figure 5:**
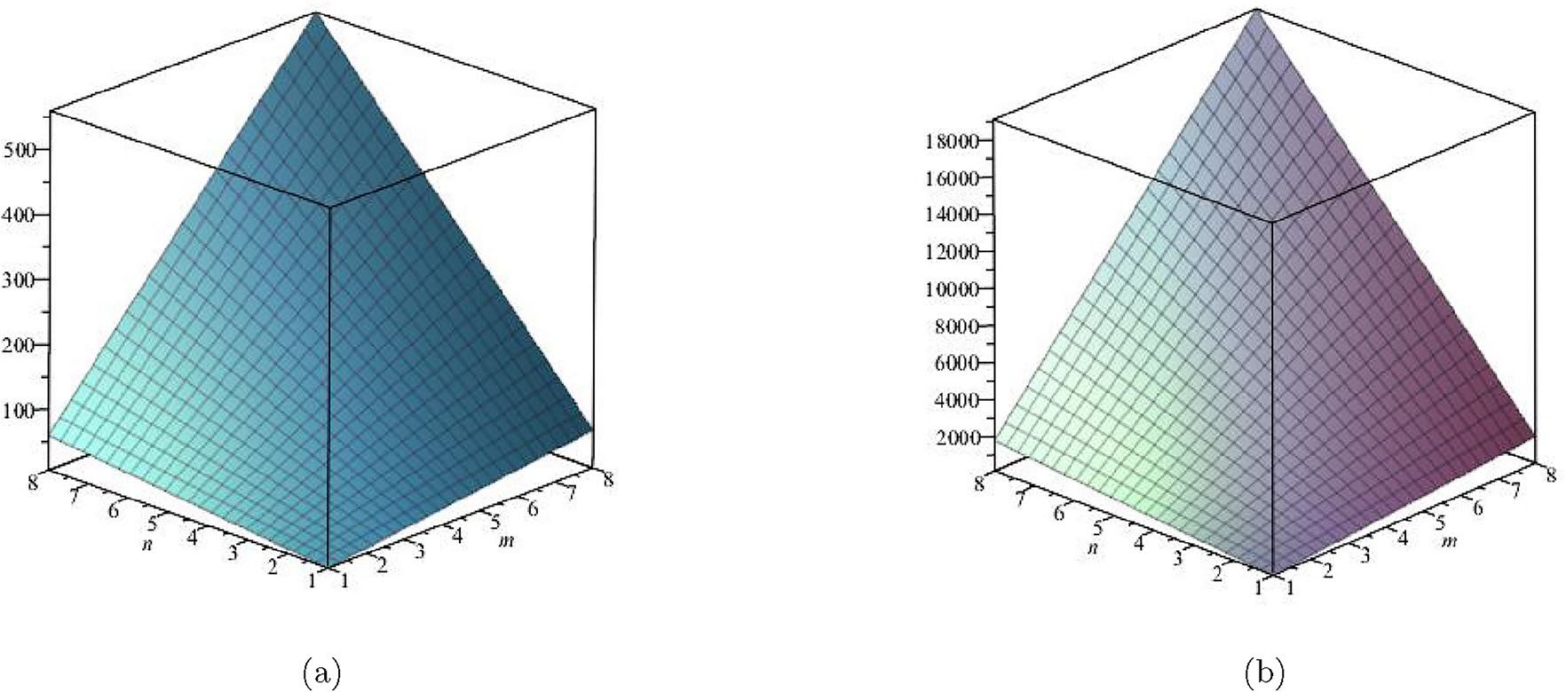
(**a**) Graph for ${R_{2}(FeTe_{2})}$,  (**b**) Graph for ${R_{-1/2}(FeTe_{2})}$.

A mathematical notion that is used to quantify the topological complexity of a graph, as shown in Figs. 4 and 5, is the Randic index. Table 3 presents a numerical comparison of the Randic indices as well.

**Table 3:** Numerical comparison of $R_1(FeTe_{2})$,  $R_{-1}(FeTe_{2})$,  $R_{\frac{1}{2}}(FeTe_{2})$,  and $R_{\frac{-1}{2}}(FeTe_{2})$.

[*m*, *n*]	$R_{1}(FeTe_{2})$	$R_{-1}(FeTe_{2})$	$R_{\frac{1}{2}}(FeTe_{2})$	$R_{\frac{-1}{2}}(FeTe_{2})$
[1, 1]	31	2.1111	14.191509	3.718744
[2, 2]	168	5.0556	64.620499	11.078917
[3, 3]	413	9.3335	151.049489	22.43909
[4, 4]	766	14.9448	273.478479	37.799263
[5, 5]	1227	21.8895	431.907469	57.159436
[6, 6]	1796	30.1676	626.336459	80.519609
[7, 7]	2473	39.7791	856.765449	107.879782
[8, 8]	3258	50.724	1123.194439	139.239955

The derivation of *ABC* index is:

$$\begin{aligned} ABC(FeTe_{2})= & {} \sum \limits _{i=1}^{5}\sum \limits _{\zeta \varpi \in E_{i}(FeTe_{2})}\sqrt{\frac{(\Upsilon (\zeta )+\Upsilon (\varpi )-2)}{\Upsilon (\zeta ) \times \Upsilon (\varpi )}}\\= & {} \sqrt{\frac{1+2-2}{(1 \times 2)}}(2)+\sqrt{\frac{1+3-2}{(1 \times 3)}}(n+1)+\sqrt{\frac{2+2-2}{(2 \times 2)}}(2m-2) \\{} & {} +\sqrt{\frac{2+3-2}{(2 \times 3)}}(4m+2n-4)+\sqrt{\frac{3+3-2}{(3 \times 3)}}(6mn-5m-3n+3)\\ ABC(FeTe_{2})= & {} 4mn + 0.909307m +0.23071n - 0.011931. \end{aligned}$$

The derivation of *GA* index is:

$$\begin{aligned} GA(FeTe_{2})= & {} \sum \limits _{i=1}^{5}\sum \limits _{\zeta \varpi \in E_{i}(FeTe_{2})}\frac{2\sqrt{\Upsilon (\zeta )\times \Upsilon (\varpi )}}{\Upsilon (\zeta )+\Upsilon (\varpi )}\\= & {} \frac{2\sqrt{1 \times 2}}{(1+2)}(2)+\frac{2\sqrt{1 \times 3}}{(1+3)}(n+1)+ \frac{2\sqrt{2 \times 2}}{(2+2)}(2m-2)\\{} & {} +\frac{2\sqrt{2 \times 3}}{(2+3)}(4m+2n-4)+\frac{2\sqrt{3 \times 3}}{(3+3)}(6mn-5m-3n+3)\\ GA(FeTe_{2})= & {} 6mn + 0.919184m - 0.174383n - 0.16754. \end{aligned}$$

The first Zagreb index :

$$\begin{aligned} M_{1}(FeTe_{2})= & {} \sum \limits _{i=1}^{5}\sum \limits _{\zeta \varpi \in E_{i}(FeTe_{2})}(\Upsilon (\zeta )+\Upsilon (\varpi ))\\= & {} (1+2)(2)+(1+3)(n+1)+(2+2)(2m-2)\\{} & {} +(2+3)(4m+2n-4)+(3+3)(6mn-5m-3n+3)\\ M_{1}(FeTe_{2})= & {} 36mn - 2m - 4n. \end{aligned}$$

The second Zagreb index:

$$\begin{aligned} M_{2}(FeTe_{2})= & {} \sum \limits _{i=1}^{5}\sum \limits _{\zeta \varpi \in E_{i}(FeTe_{2})}(\Upsilon (\zeta )\times \Upsilon (\varpi ))\\= & {} (1 \times 2)(2)+(1 \times 3)(n+1)+(2 \times 2)(2m-2)\\{} & {} +(2 \times 3)(4m+2n-4)+(3 \times 3)(6mn-5m-3n+3)\\ M_{2}(FeTe_{2})= & {} 54mn - 13m - 12n + 2.\\ \end{aligned}$$

**Figure 6:**
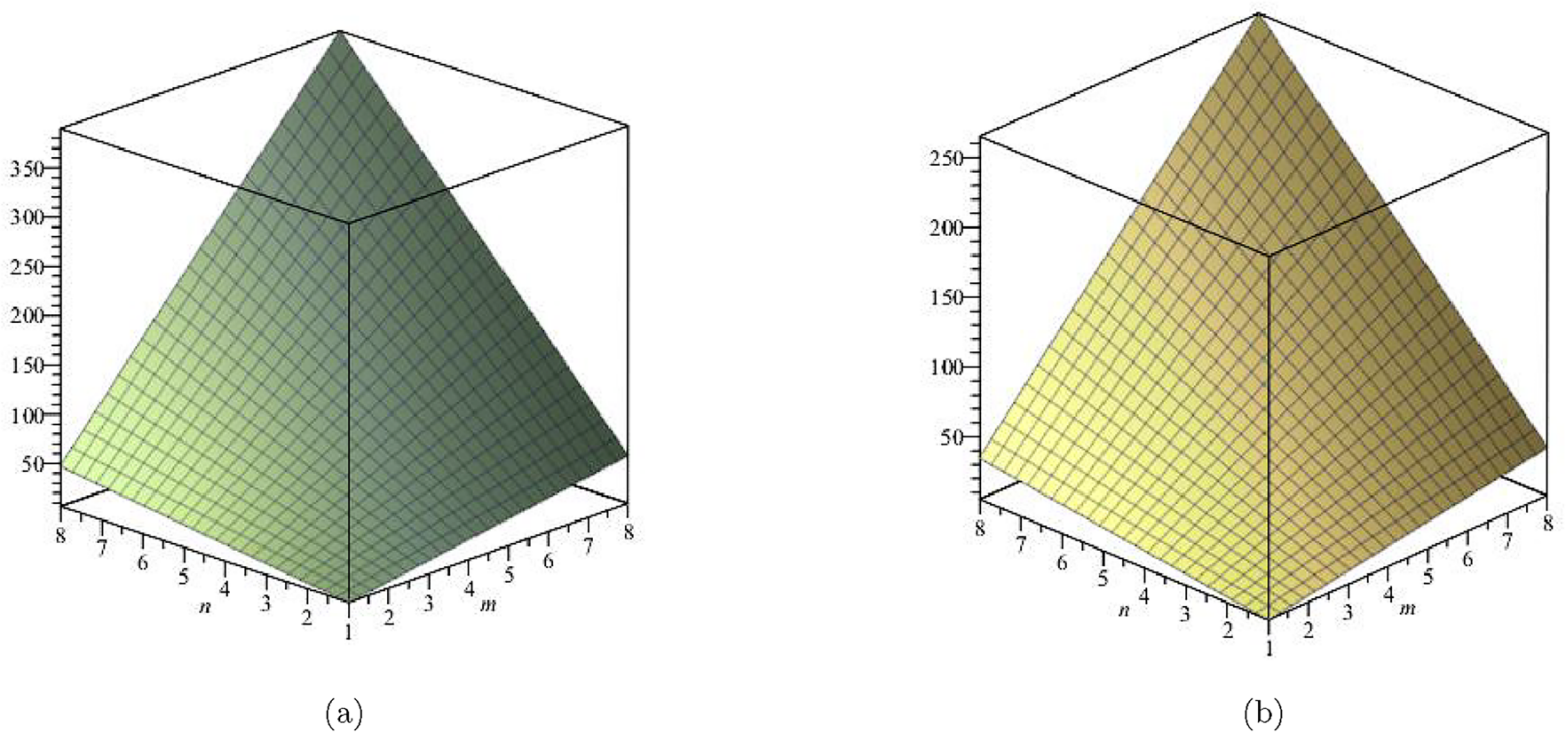
(**a**) Graph for ${ABC(FeTe_{2})}$, (**b**) Graph for ${GA(FeTe_{2})}$.

**Figure 7:**
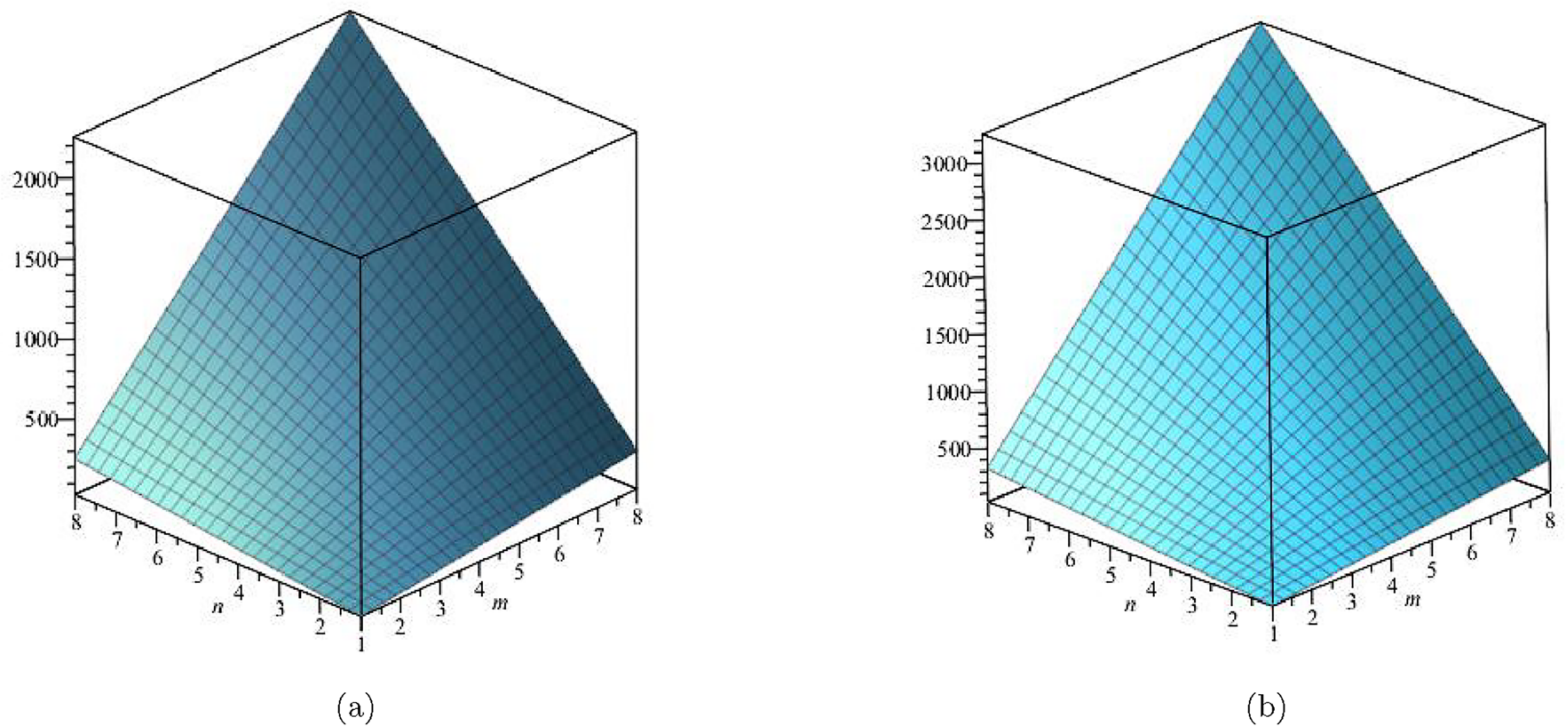
(**a**) Graph for ${M_{1}(FeTe_{2})}$,  (**b**) Graph for ${M_{2}(FeTe_{2})}$.

The numerical comparison and graphical depiction of the $ABC(FeTe_2)$, $GA(FeTe_2)$, $M_1(FeTe_2)$, and $M_2(FeTe_2)$, respectively, are shown in Table 4 and Figs. 6 and 7.

**Table 4:** Numerical Comparison of $ABC(FeTe_{2})$,  $GA(FeTe_{2})$,  $M_{1}(FeTe_{2})$ and $M_{2}(FeTe_{2})$.

[*m*, *n*]	$ABC(FeTe_{2})$	$GA(FeTe_{2})$	$M_1(FeTe_{2})$	$M_2(FeTe_{2})$
[1, 1]	5.128086	6.577261	30	31
[2, 2]	18.268103	25.322062	132	168
[3, 3]	39.40812	56.066863	306	413
[4, 4]	68.548137	98.811664	552	766
[5, 5]	150.688154	153.556465	870	1227
[6, 6]	150.828171	220.301266	1260	1796
[7, 7]	203.968188	299.046067	1722	2473
[8, 8]	265.108205	389.790868	2256	3258

The Hyper Zagreb index:

$$\begin{aligned} HM(FeTe_{2})= & {} \sum \limits _{i=1}^{5}\sum \limits _{\zeta \varpi \in E_{i}(FeTe_{2})}(\Upsilon (\zeta )+\Upsilon (\varpi ))^{2}\\= & {} (1+2)^{2}(2)+(1+3)^{2}(n+1)+(2+2)^{2}(2m-2)\\{} & {} +(2+3)^{2}(4m+2n-4)+(3+3)^{2}(6mn-5m-3n+3)\\ HM(FeTe_{2})= & {} 216mn - 48m - 42n +10. \end{aligned}$$

The derivation of the Forgotten index:

$$\begin{aligned} F(FeTe_{2})= & {} \sum \limits _{i=1}^{5}\sum \limits _{\zeta \varpi \in E_{i}(FeTe_{2})}(\Upsilon (\zeta )^{2}+\Upsilon (\varpi )^{2})\\= & {} (1^{2}+2^{2})(2)+(1^{2}+3^{2})(n+1)+(2^{2}+2^{2})(2m-2)\\{} & {} +(2^{2}+3^{2})(4m+2n-4)+(3^{2}+3^{2})(6mn-5m-3n+3)\\ F(FeTe_{2})= & {} 108mn - 22m - 18n + 6. \end{aligned}$$

The derivation of the Augmented Zagreb index:

$$\begin{aligned} AZI(FeTe_{2})= & {} \sum \limits _{i=1}^{5}\sum \limits _{\zeta \varpi \in E_{i}(FeTe_{2})}\left[ \frac{(\Upsilon (\zeta )\times \Upsilon (\varpi ))}{\Upsilon (\zeta )+\Upsilon (\varpi )-2}\right] ^{3}\\= & {} \Big (\frac{1 \times 2}{1+2-2}\Big )^{3}(2)+\Big (\frac{1 \times 3}{1+3-2}\Big )^{3}(n+1)+\Big (\frac{2 \times 2}{2+2-2}\Big )^{3}(2m-2)\\{} & {} +\Big (\frac{2 \times 3}{2+3-2}\Big )^{3}(4m+2n-4)+\Big (\frac{3 \times 3}{3+3-2}\Big )^{3}(6mn-5m-3n+3)\\ AZI(FeTe_{2})= & {} 68.34375mn - 8.953125m - 14.796875n + 5.546875. \end{aligned}$$

The Balaban index:

$$\begin{aligned} J(FeTe_{2})= & {} \left( \frac{\acute{r}}{\acute{r}-\acute{s}+2}\right) \bigg [\sum _{i=1}^{5}\sum \limits _{\zeta \varpi \in E_{i}(FeTe_{2})}\frac{1}{\sqrt{\Upsilon (\zeta ) \times \Upsilon (\varpi )}}\bigg ]\\= & {} \left( \frac{6mn+m}{(6mn+m)-(4mn+2m+n+1)+2}\right) \bigg [\frac{2}{\sqrt{(1 \times 2)}}+\frac{n+1}{\sqrt{(1 \times 3)}}\\{} & {} +\frac{2m-2}{\sqrt{(2 \times 2)}}+\frac{4m+2n-4}{\sqrt{(2 \times 3)}}+\frac{6mn-5m-3n+3}{\sqrt{(3 \times 3)}}\bigg ]\\ J(FeTe_{2})= & {} \left( \frac{6mn+m}{2mn-m-n+1}\right) (2mn + 0.966326m + 0.393847n + 0.358571).\\ \end{aligned}$$

**Figure 8:**
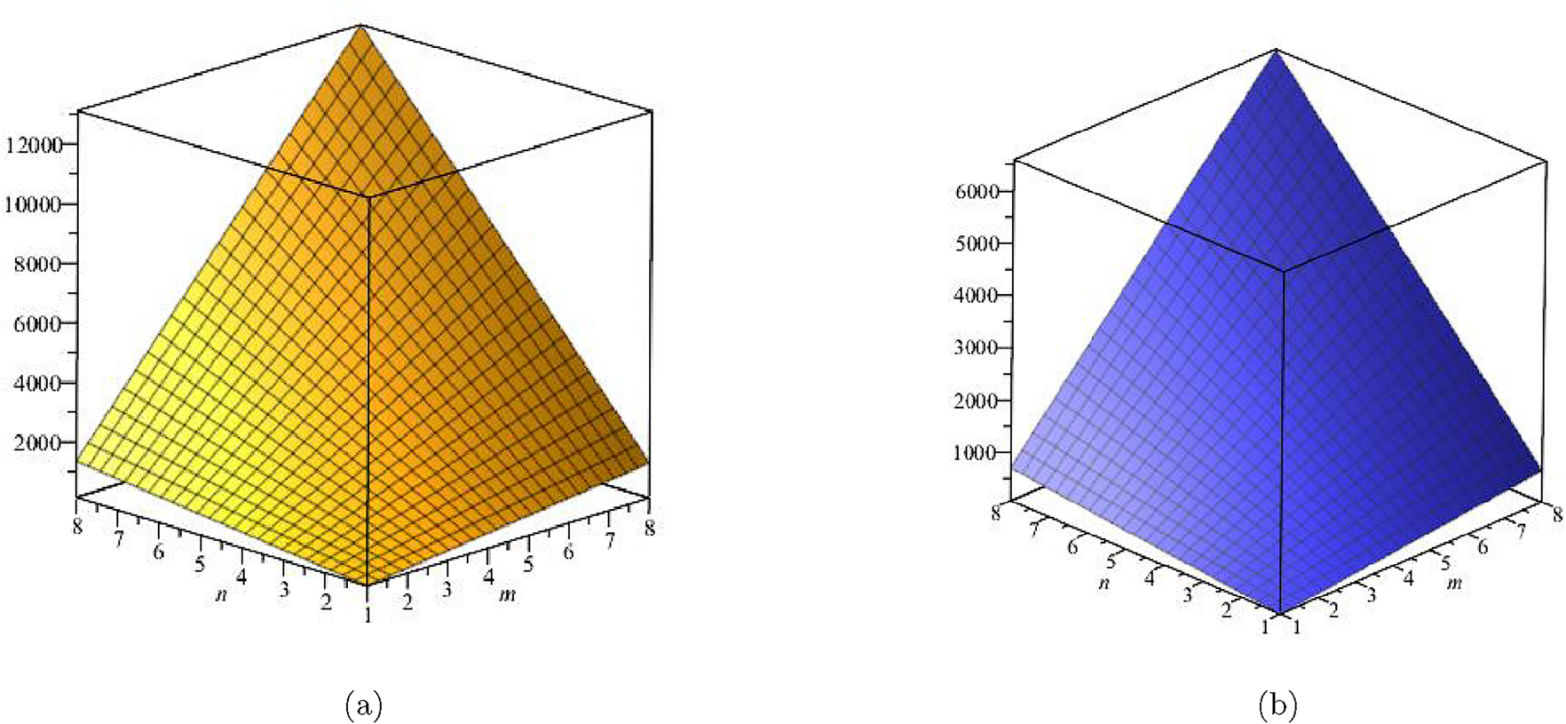
(**a**) Graph for ${HM(FeTe_{2})}$,  (**b**) Graph for ${F(FeTe_{2})}$.

**Figure 9:**
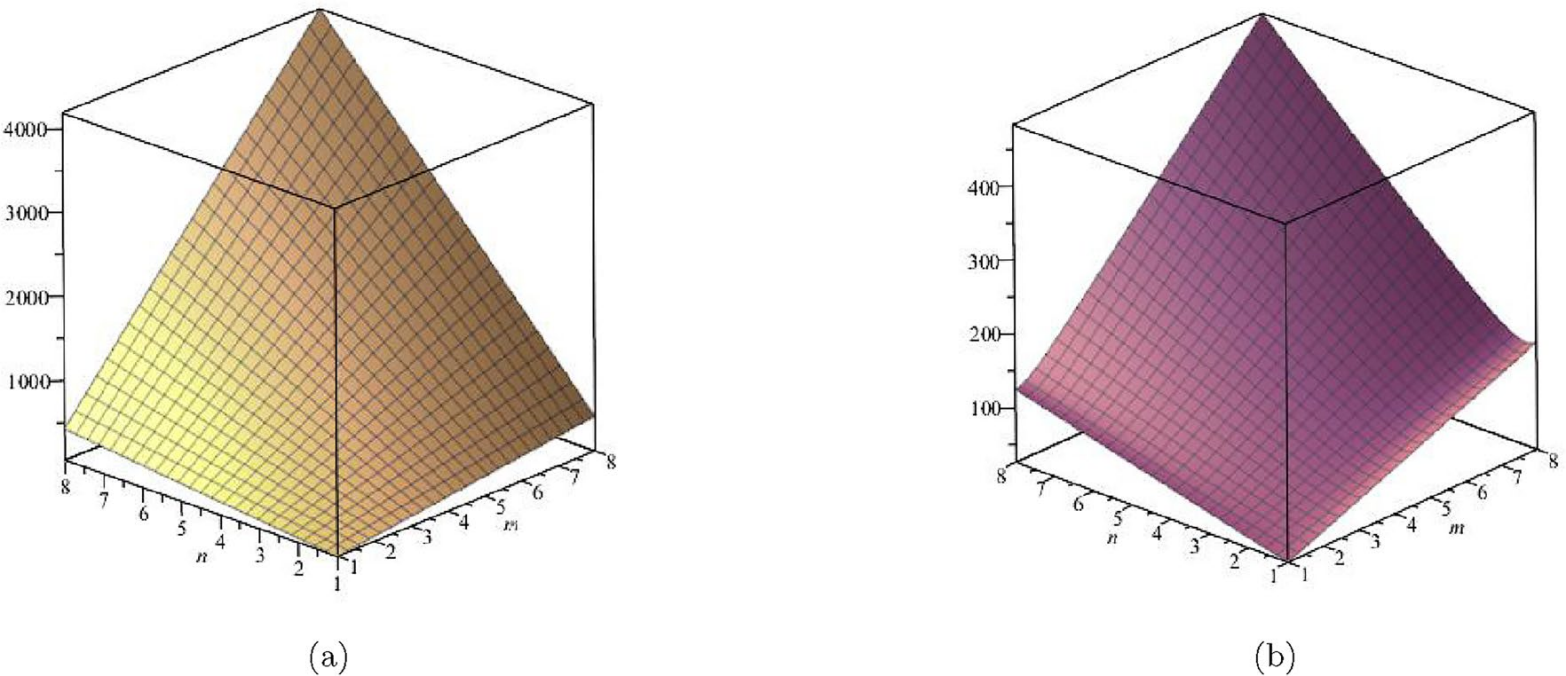
(**a**) Graph for ${AZI(FeTe_{2})}$,  (**b**) Graph for ${J(FeTe_{2})}$.

The *HM*, *F*, *AZI*, and *J*, respectively, are represented graphically and numerically in Table 5 and Figs. 8 and 9.

**Table 5:** Numerical pattern of $HM(FeTe_{2})$,  $F(FeTe_{2})$,  $AZI(FeTe_{2})$ and $J(FeTe_{2})$.

[*m*, *n*]	$HM(FeTe_{2})$	$F(FeTe_{2})$	$AZI(FeTe_{2})$	$J(FeTe_{2})$
[1, 1]	136	74	50.140625	26.031208
[2, 2]	694	358	231.421875	57.6103684
[3, 3]	1684	858	549.390625	98.386779
[4, 4]	3106	1574	1004.046875	151.197052
[5, 5]	4960	2506	1595.390625	216.0905507
[6, 6]	7246	3654	2323.421875	293.038577
[7, 7]	9964	5018	3188.140625	382.0213457
[8, 8]	13,114	6598	4189.546875	483.027101

The initial multiple Zagreb index was identified in our investigation as:

$$\begin{aligned} PM_{1}(FeTe_{2})= & {} \prod \limits _{i=1}^{5}\prod \limits _{\zeta \varpi \in E_{i}(FeTe_{2})}(\Upsilon (\zeta )+\Upsilon (\varpi ))\\= & {} (1+2)\times (2)\times (1+3)\times (n+1)\times (2+2)\times (2m-2)\\{} & {} \times (2+3)\times (4m+2n-4)\times (3+3)\times (6mn-5m-3n+3)\\ PM_{1}(FeTe_{2})= & {} (192mn + 192m - 192n - 192)(20m + 10n - 20)(36mn - 30m - 18n + 18). \end{aligned}$$

We defined the second multiple Zagreb index in our investigation as:

$$\begin{aligned} PM_{2}(FeTe_{2})= & {} \prod \limits _{i=1}^{5}\prod \limits _{\zeta \varpi \in E_{i}(G)}(\Upsilon (\zeta )\times \Upsilon (\varpi ))\\= & {} (1 \times 2)\times (2)\times (1\times 3)\times (n+1) \times (2 \times 2)\times (2m-2)\\{} & {} \times (2 \times 3)\times (4m+2n-4)\times (3 \times 3)\times (6mn-5m-3n+3)\\ PM_{2}(FeTe_{2})= & {} (96mn + 96m - 96n - 96)(24m + 12n - 24)(54mn - 45m - 27n + 27).\\ \end{aligned}$$

**Figure 10:**
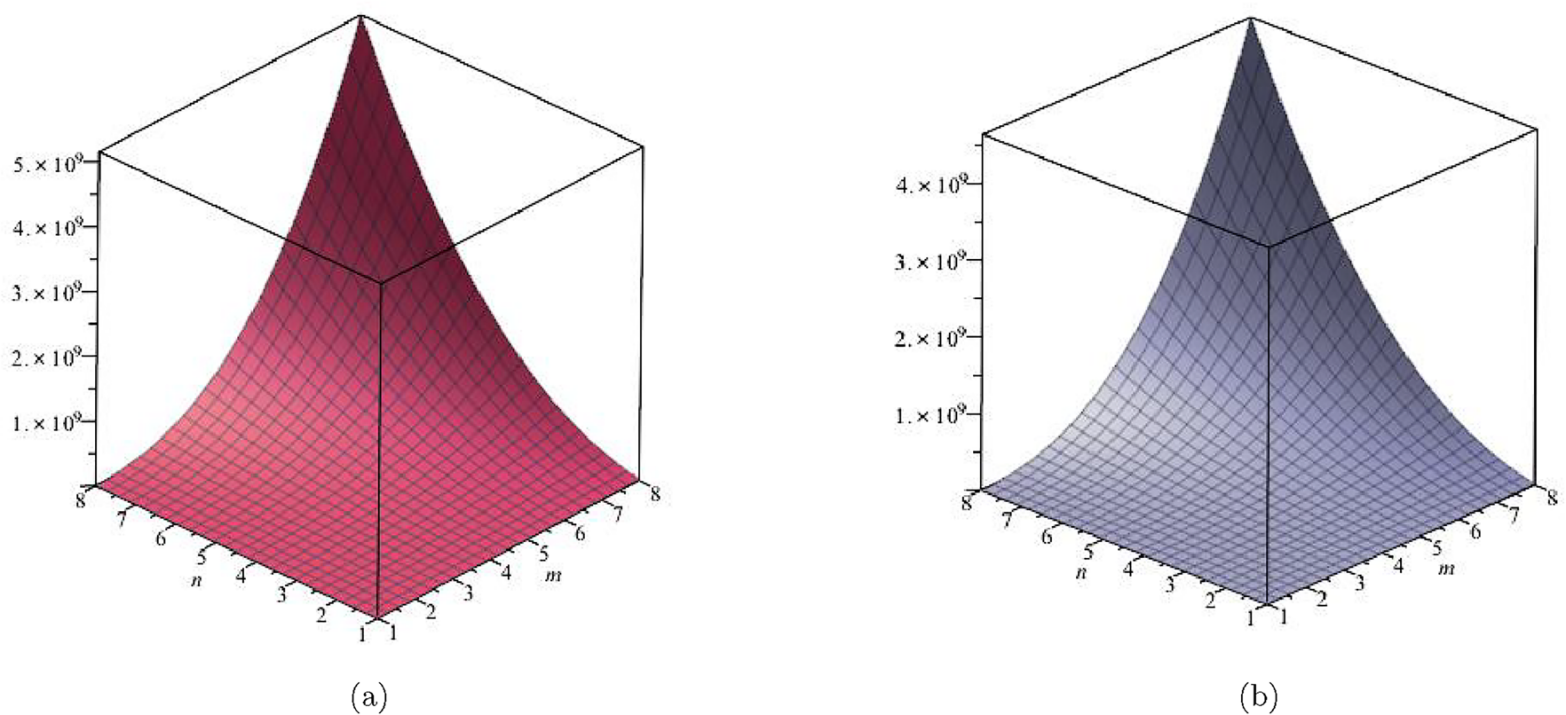
(**a**) Graph for ${PM_{1}(FeTe_{2})}$,  (**b**) Graph for ${PM_{2}(FeTe_{2})}$.

The $PM_1$ and $PM_2$ are represented graphically and numerically in Table 6 and Fig. 10.

**Table 6:** Numerical pattern of $PM_{1}(FeTe_{2})$,  $PM_{2}(FeTe_{2})$.

[*m*, *n*]	$PM_{1}(FeTe_{2})$	$PM_{2}(FeTe_{2})$
[2, 2]	1,520,640	1,368,576
[3, 3]	21,288,960	19,160,064
[4, 4]	115,776,000	104,198,400
[5, 5]	406,149,120	365,534,208
[6, 6]	1,103,155,200	992,839,680
[7, 7]	2,532,003,840	2,278,803,456
[8, 8]	5,157,250,560	4,641,525,504

## Analysis of heat of formation via rational curve fitting

A mathematical model’s parameters are found using a statistical technique called curve fitting, which enables the model to approximately describe the observed data. In this section, we give the concept of the Information entropy and Heat of Formation(Enthalpy) of Iron Telluride $(FeTe_2)$. The standard molar enthalpy of Iron Telluride $(FeTe_2)$ or $(FeTe_{1.99})$ is $-51.9(-65.8) kJ/mol$ at temperature 298.15*K*. Mathematical formula to calculate Heat of Formation (HOF) for different formula units is given as:

12 $$\begin{aligned} HOF = \frac{Standard \,\,\ Molar\,\,\ HOF}{Avogadro's\,\,\ Number}\,\,\ \times \,\,\ Formula\,\,\ Units \end{aligned}$$

where, $Avogadro's Number = 6.02214076 \times 10^{23} mol^{-1}$. A substance’s mole is equivalent to $6.02214076 \times 10^{23} mol^{-1}$ of that material (such as atoms, molecules, or ions).Each material is in its normal state when a mole of a chemical is created by combining its constituent elements, and the amount of energy absorbed or released is measured by the heat of formation (*HOF*). This is measured in kilojoules per mole, or *KJ*/*mol*. It can also be described as having a normal heat of formation or a specific heat capacity.

**Figure 11:**
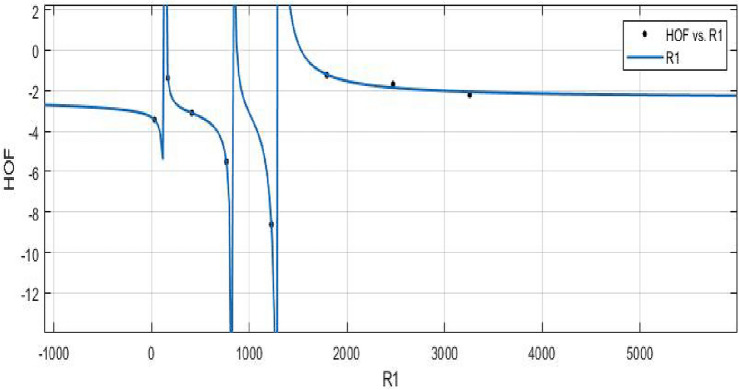
$\mathfrak {R}_{{c}{f}}$ between HOF of $R_{1}(FeTe_{2})$.

**Figure 12:**
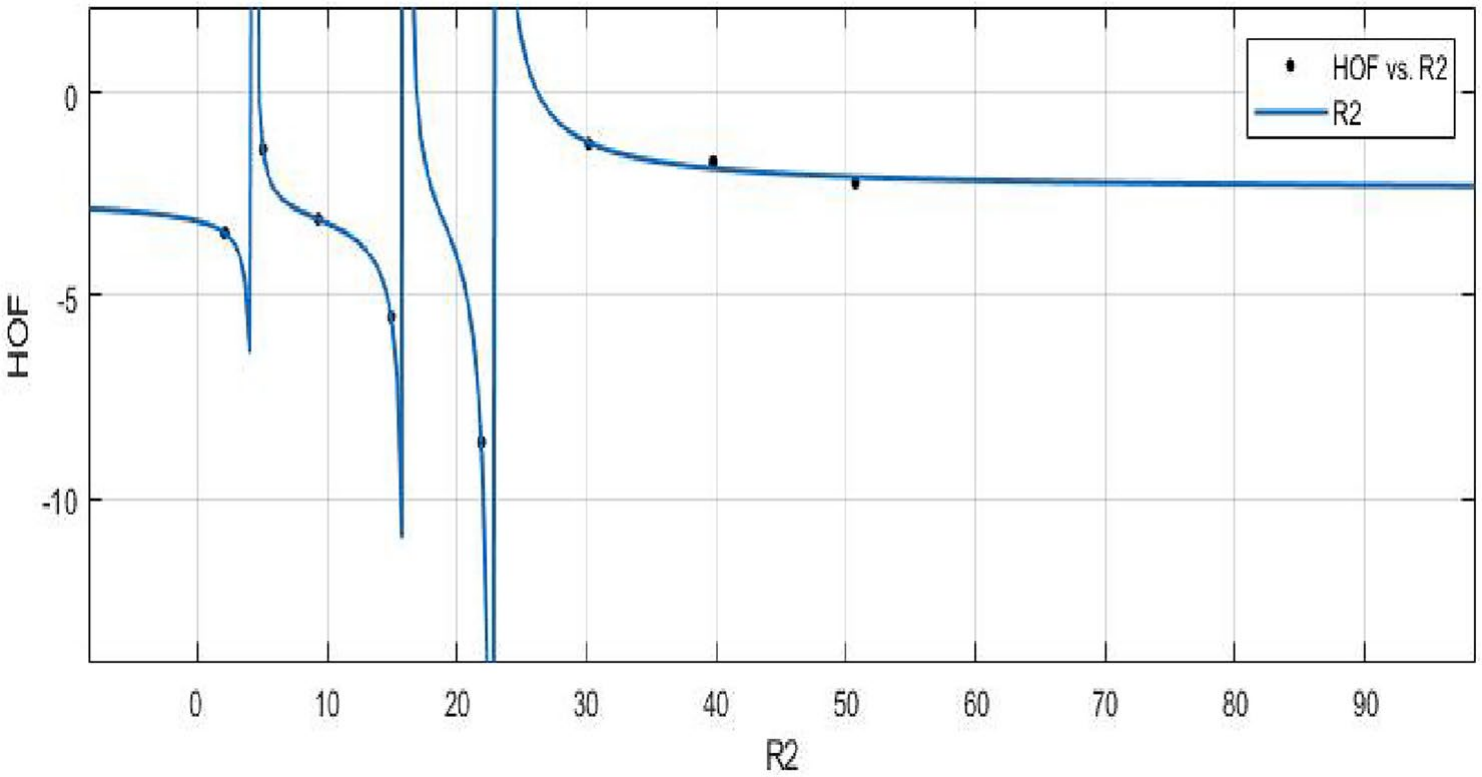
$\mathfrak {R}_{{c}{f}}$ between HOF of $R_{-1}(FeTe_{2})$.

**Figure 13:**
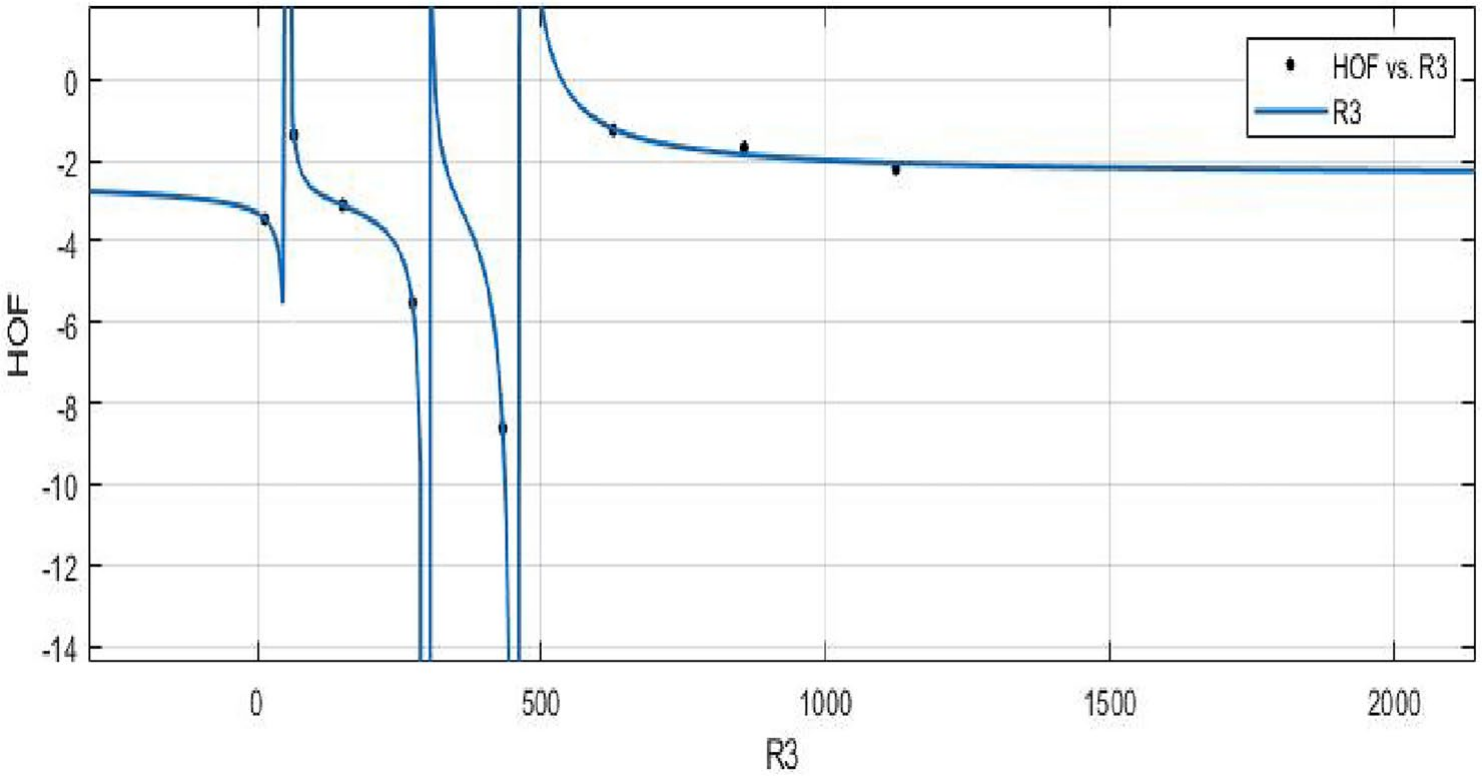
$\mathfrak {R}_{{c}{f}}$ between HOF of ${R_{\frac{1}{2}}(FeTe_{2})}$.

**Figure 14:**
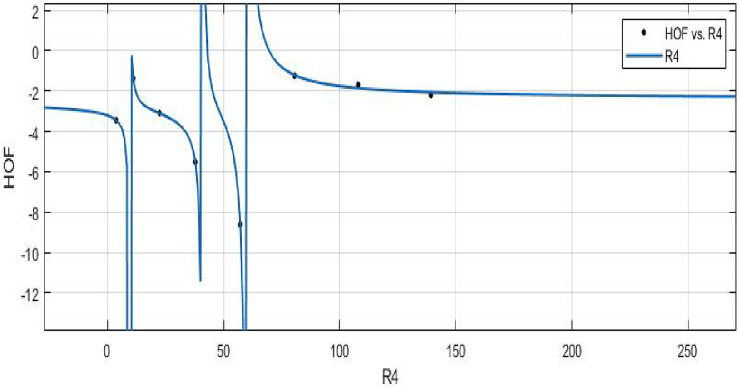
$\mathfrak {R}_{{c}{f}}$ between HOF of $R_{\frac{-1}{2}}(FeTe_{2})$.

**Figure 15:**
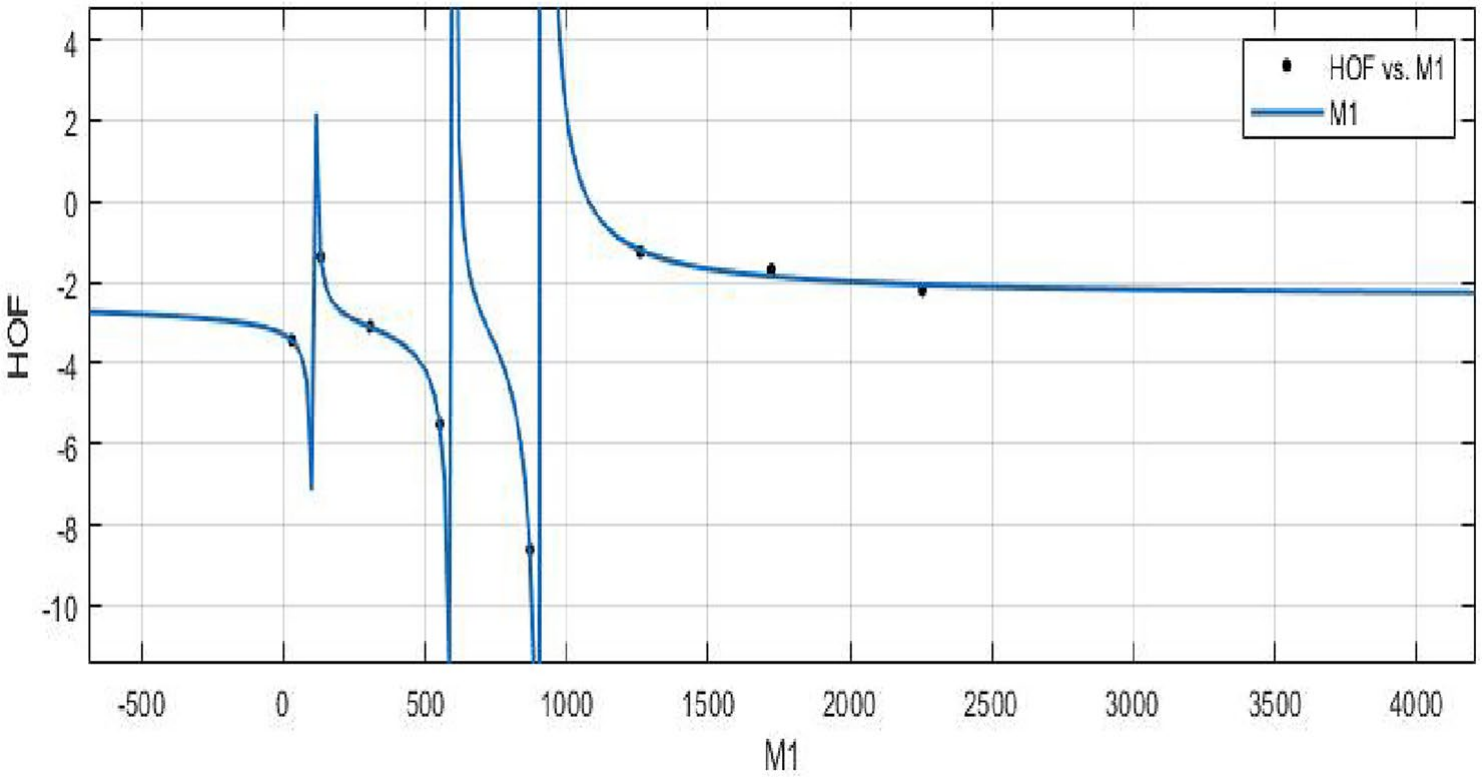
$\mathfrak {R}_{c}{f}$ between HOF of $M_{1}(FeTe_{2})$.

**Figure 16:**
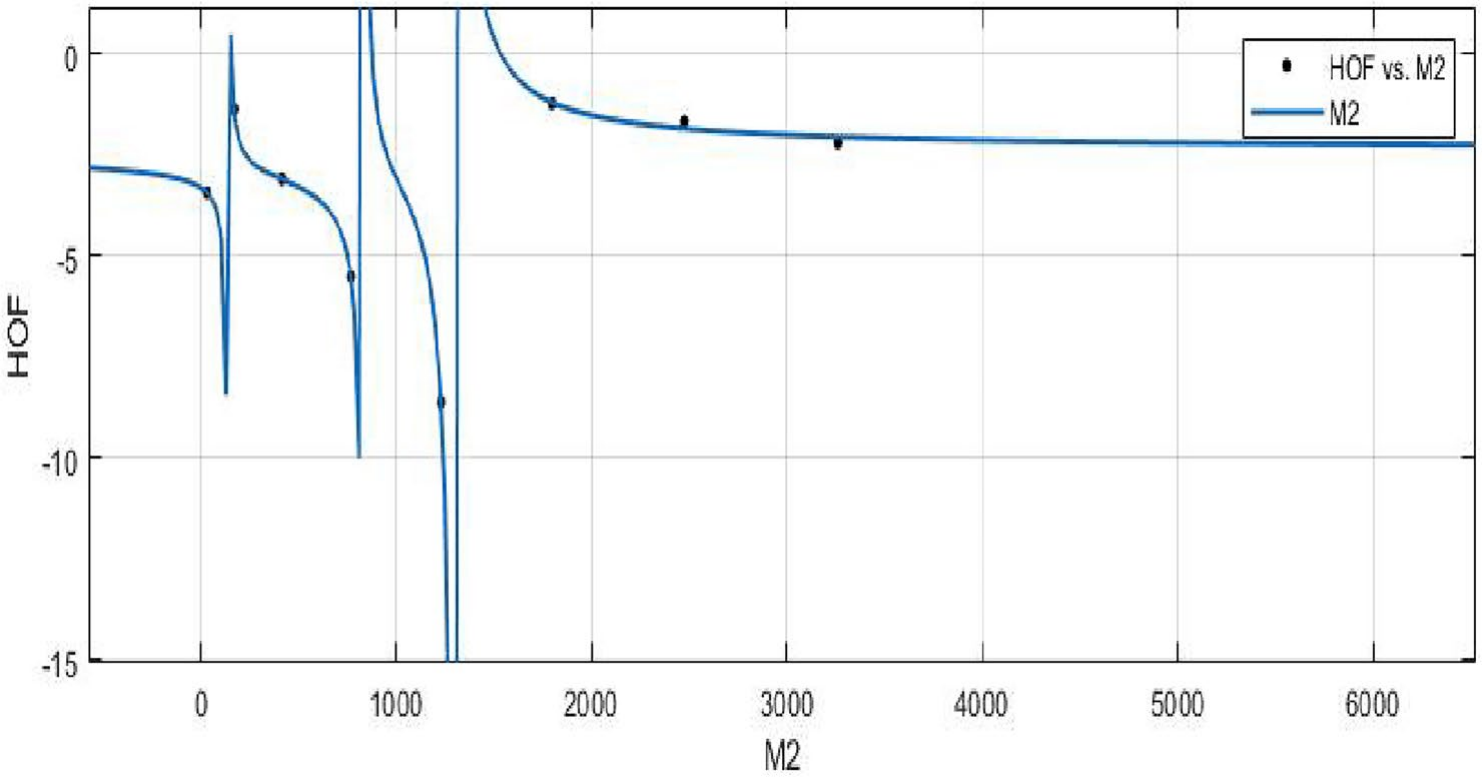
$$\mathfrak {R}_{c}{f}$$ between *HOF* of $$ M_{2}(FeTe_{2})$$.

**Figure 17:**
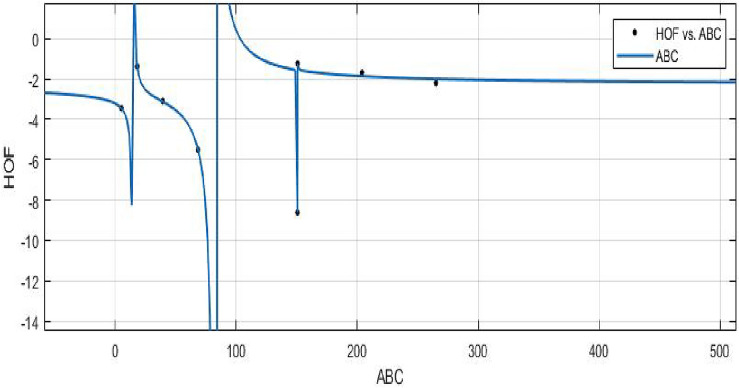
$\mathfrak {R}_{c}{f}$ between HOF of ${ABC}(FeTe_{2})$.

**Figure 18:**
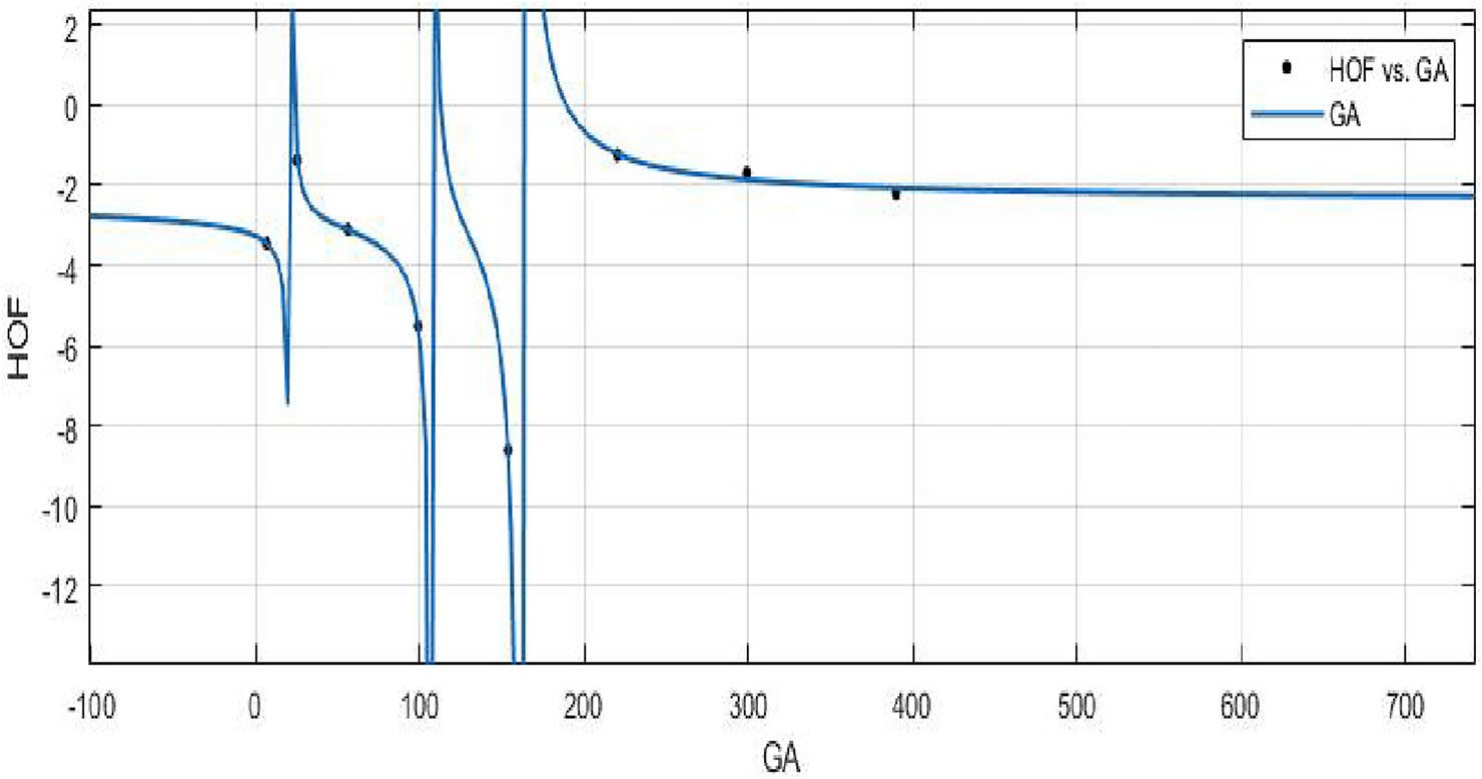
$\mathfrak {R}_{c}{f}$ between HOF of $GA(FeTe_{2})$.

**Figure 19:**
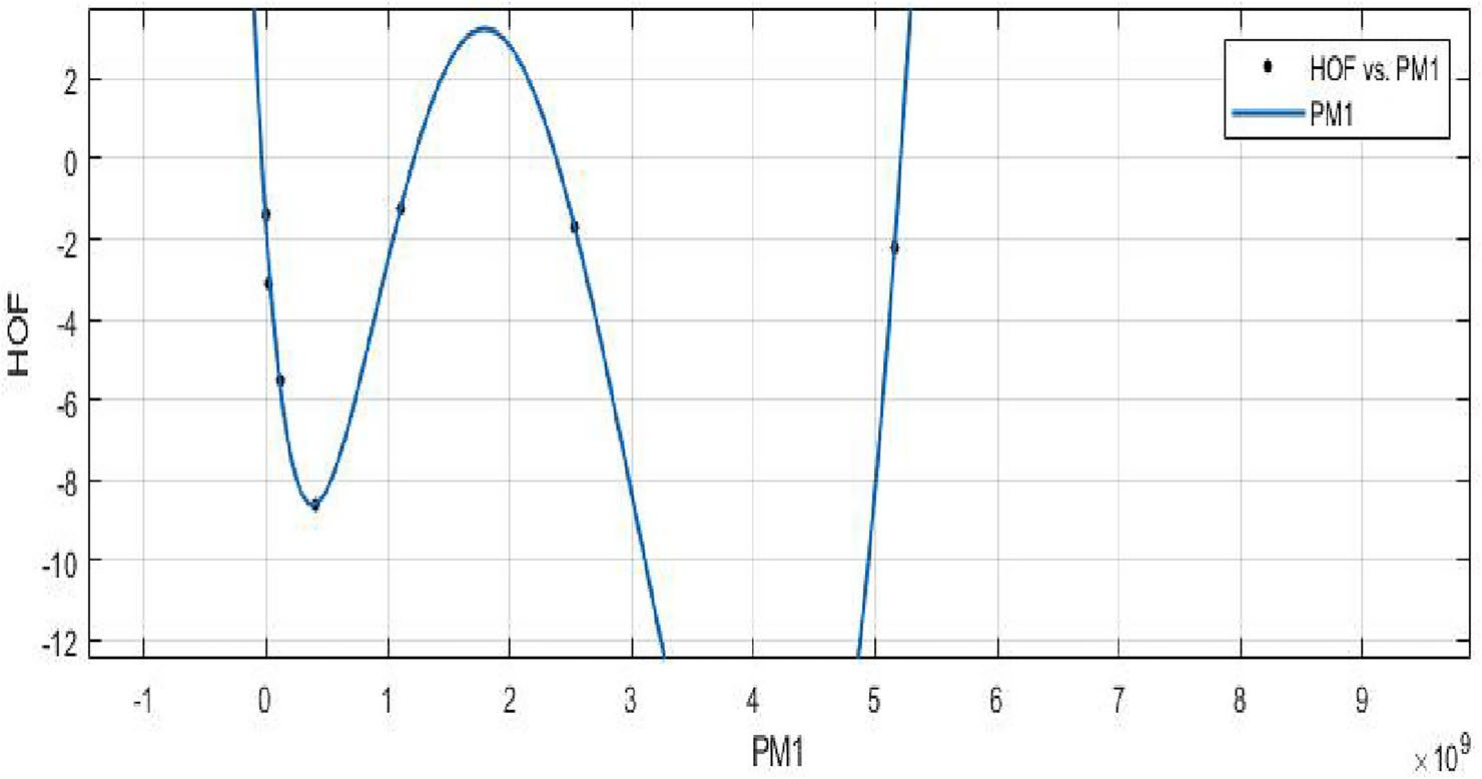
$\mathfrak {R}_{c}{f}$ between *HOF* of ${PM_1}(FeTe_{2})$.

**Figure 20:**
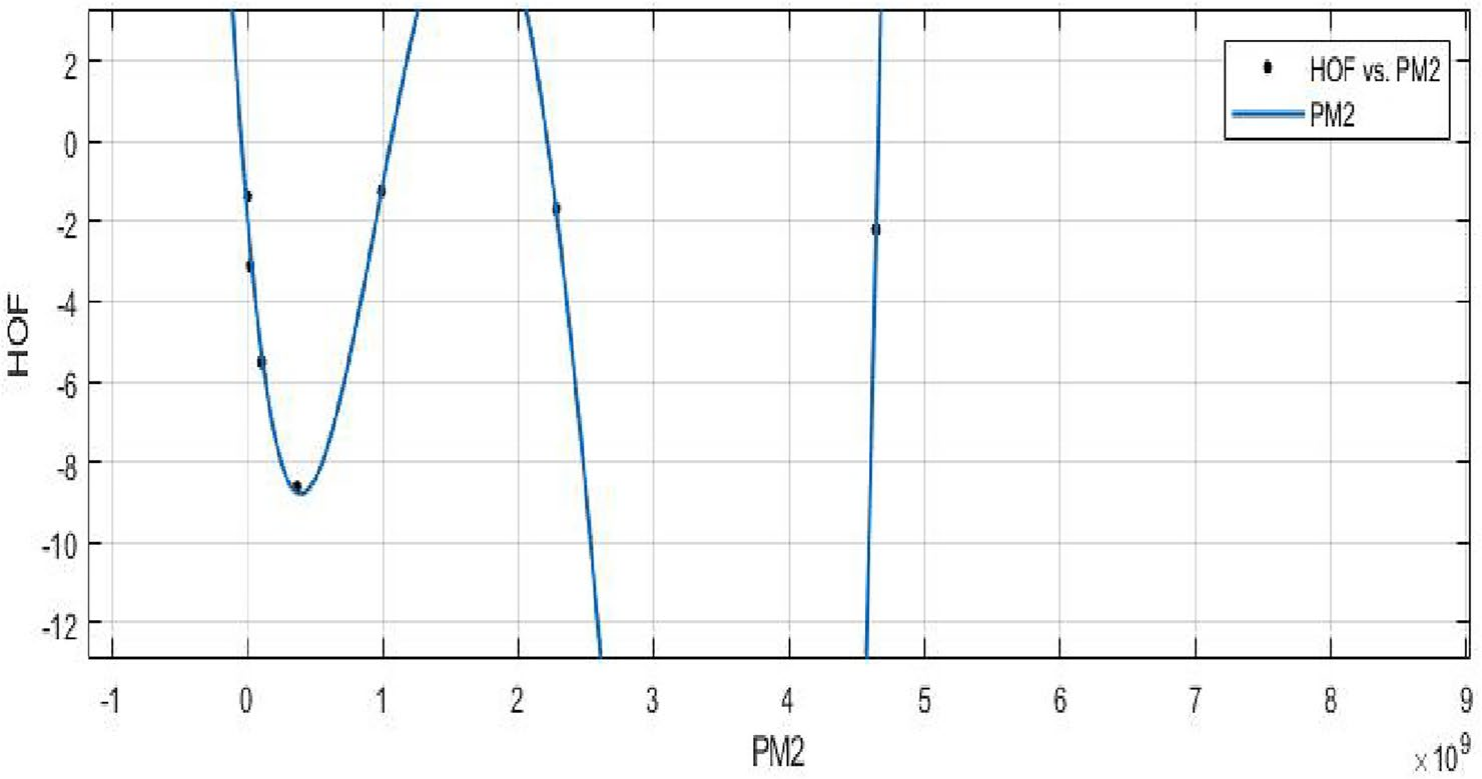
$\mathfrak {R}_{c}{f}$ between *HOF* of $PM_{2}(FeTe_{2})$.

**Figure 21:**
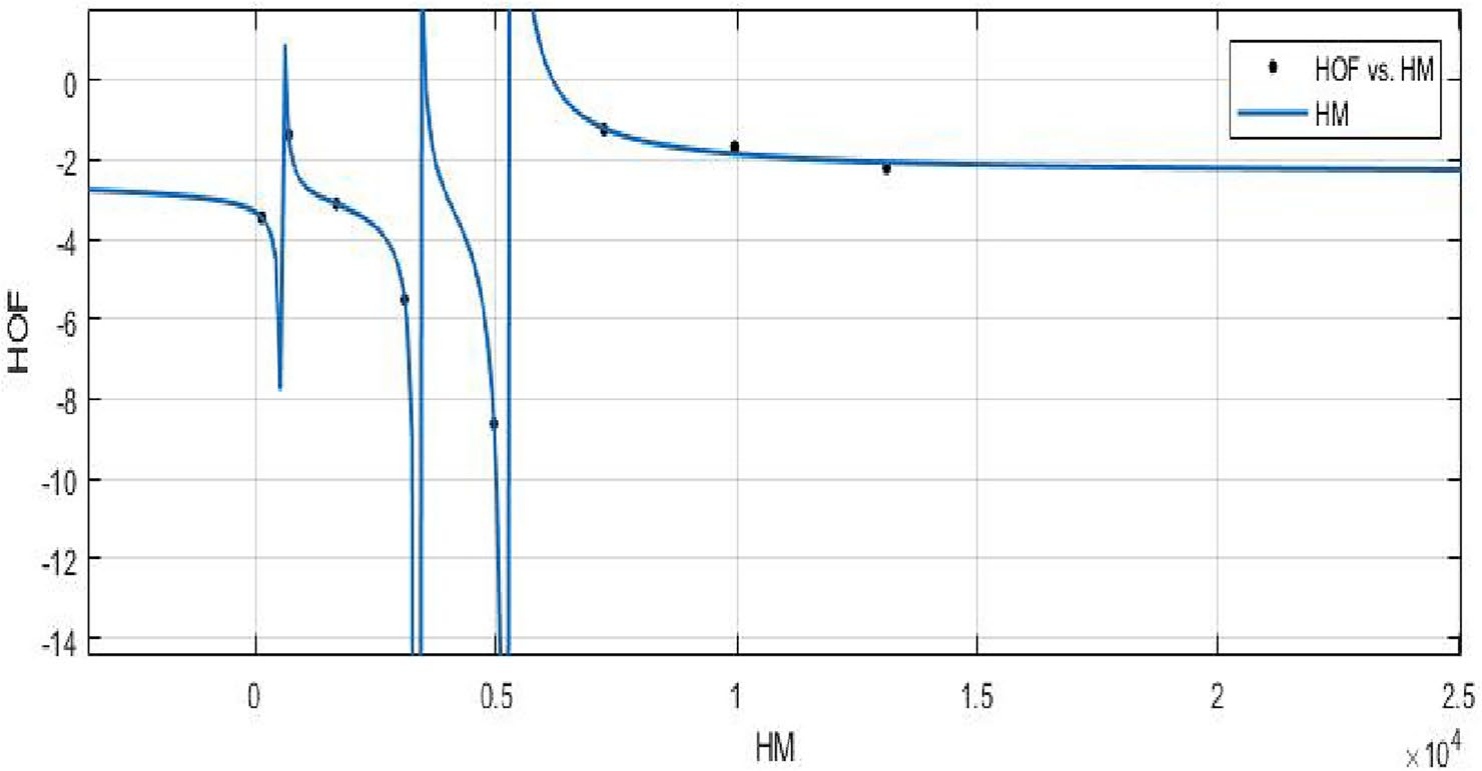
$\mathfrak {R}_{c}{f}$ between *HOF* of ${HM}(FeTe_{2})$.

**Figure 22:**
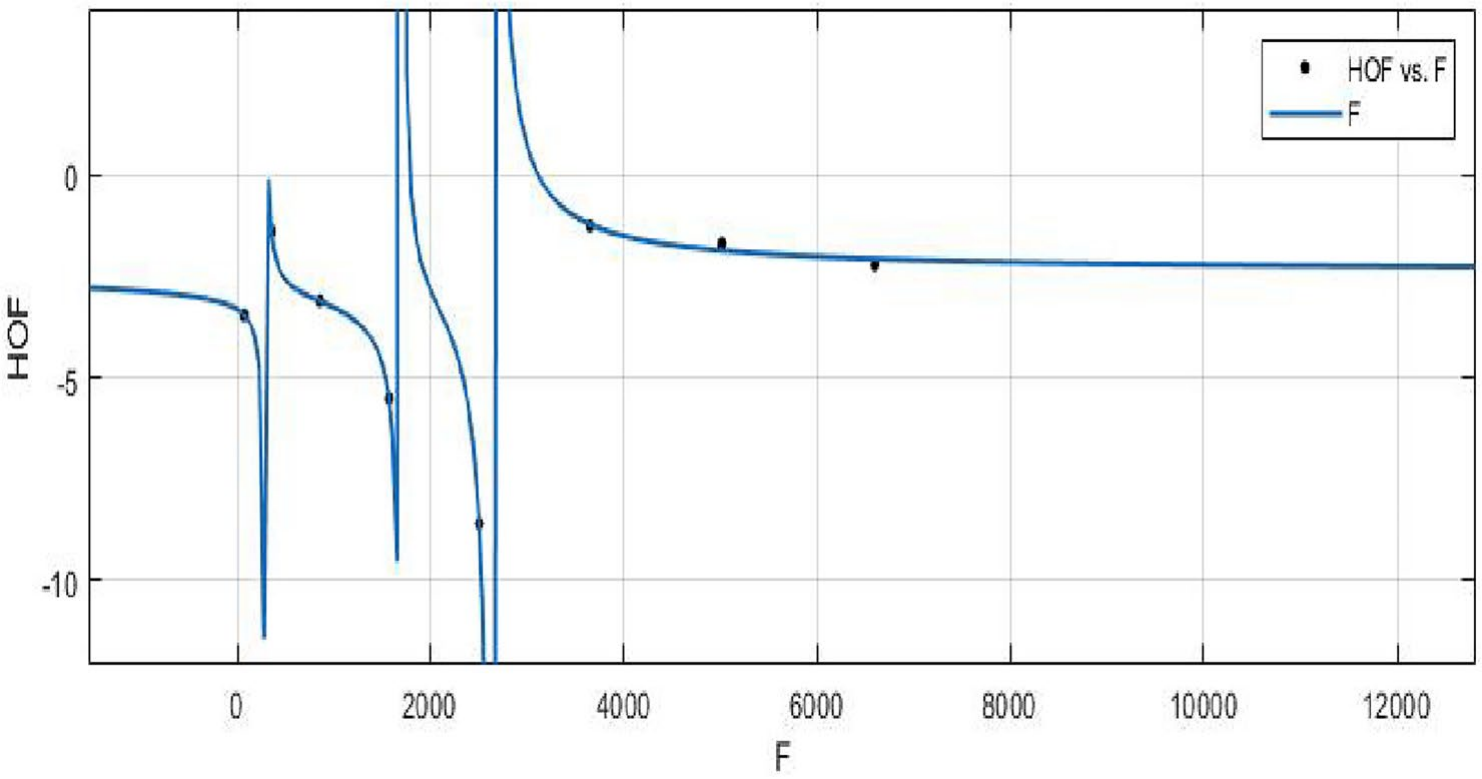
$\mathfrak {R}_{c}{f}$ between *HOF* of ${F}(FeTe_{2})$.

**Figure 23:**
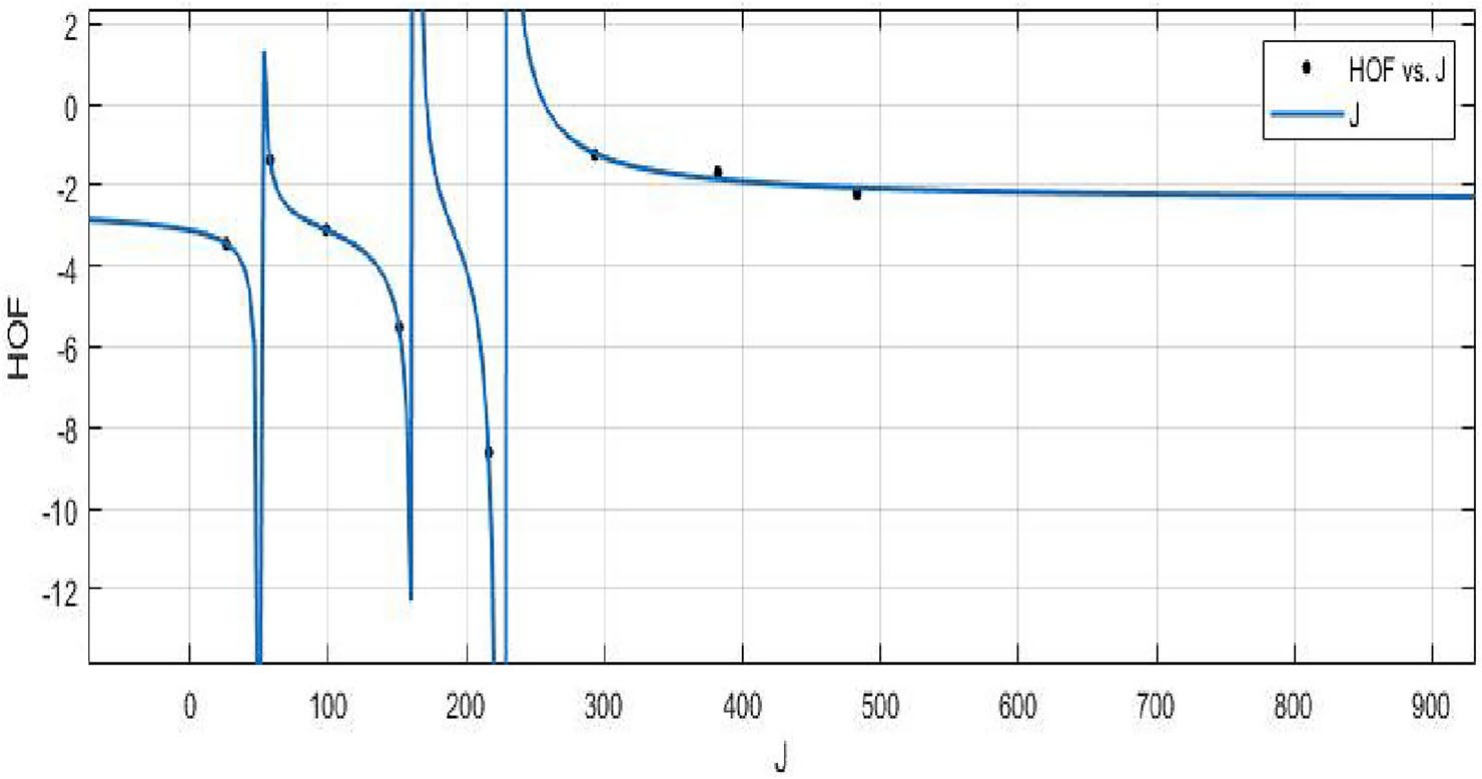
$\mathfrak {R}_{c}{f}$ between *HOF* of ${J}(FeTe_{2})$.

**Figure 24:**
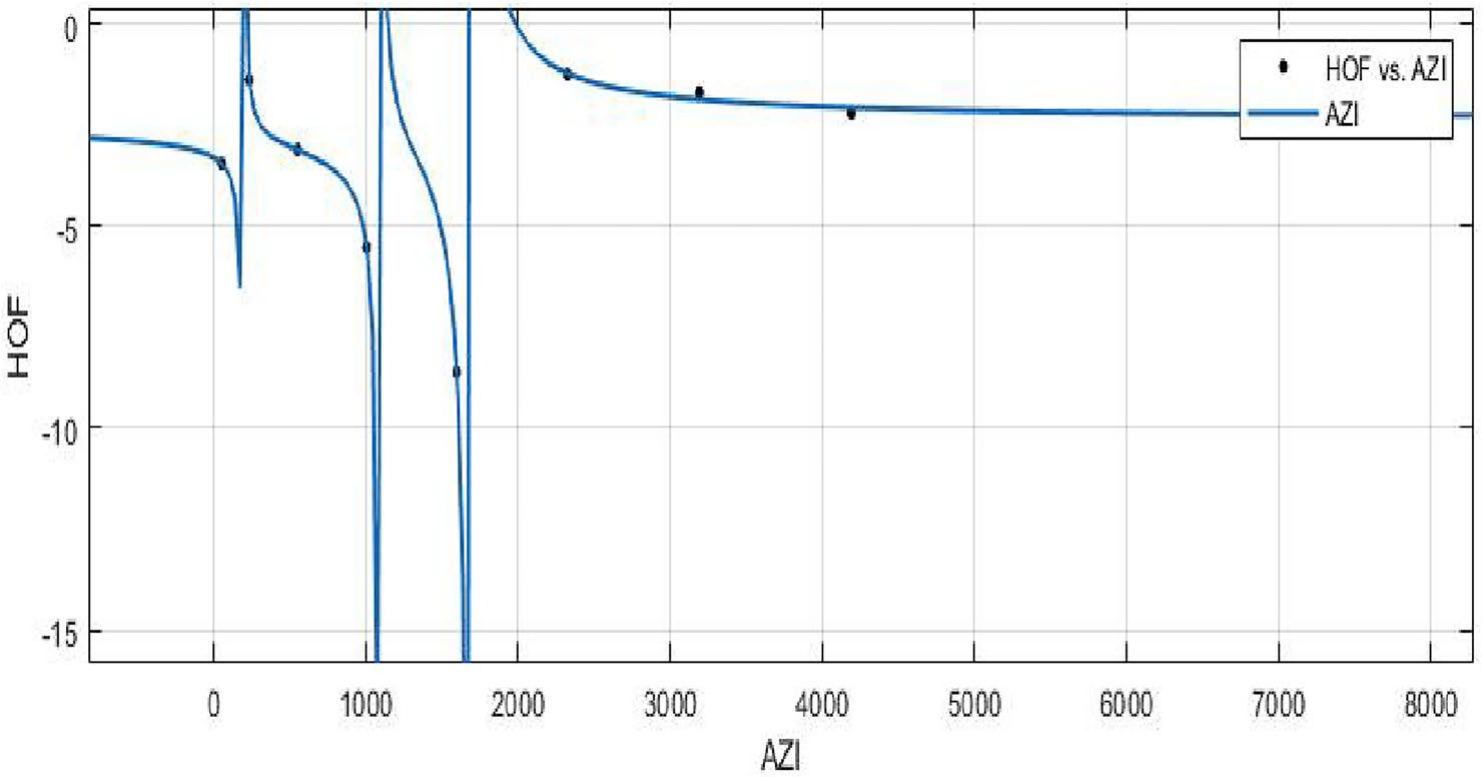
$\mathfrak {R}_{c}{f}$ between *HOF* of ${AZI}(FeTe_{2})$.

MATLAB’s Power built-in function is used to create models between Heat of Formation and each information entropy since it provides a lower *RMSE* value for the best fit. The mean squared error (*RMSE*), the sum of squared errors (*SSE*), and $R^2$ are the accuracy metrics that are employed. First, calculate the values of various indices using the Shanon entropy formula. Then we use the Maple software to determine the entropy’s graphical behavior and computed numerical values. Furthermore, curves were fitted between the Heat of Formation values and a number of related entropies. The models for the relationship between indices vs. *HOF* that were described are shown below. Graphical behaviour is presented in Figs. 11, 12, 13, 14, 15, 16, 17, 18, 19, 20, 21, 22, 23, 24.

- **HOF using** $$R_1(FeTe_{2})$$

$$\begin{aligned} f(R_1) = \frac{\left( \chi _1\times R_1^3 + \chi _2\times R_1^2 + \chi _3\times R_1 + \chi _4\right) }{ \left( R_1^3 + \Upsilon _1\times R_1^2 + \Upsilon _2\times R_1 + \Upsilon _3\right) } \end{aligned}$$

$R_1$ is standardized(normalized) by using the symbol $\mu$ (*mu*) to represent the mean value of 1267 and the symbol $\sigma$ (*sigma*) to represent the standard deviation of 1160. The Coefficients: $\chi _1 = -2.389$, with $\mathbb {C}_{b} = (-4.81, 0.03131)$, $\chi _2= -2.559$, with $\mathbb {C}_{b} = (-6.793, 1.674)$, $\chi _3 = -0.07126$, with $\mathbb {C}_{b} = (-1.813, 1.671)$, $\chi _4 = 0.1775$, with $\mathbb {C}_{b} = (-1.633, 1.988)$, $\Upsilon _1 = 1.32$, with $\mathbb {C}_{b} = (0.7449, 1.896)$, $\Upsilon _2 = 0.3262$, with $\mathbb {C}_{b} = (-0.08985, 0.7423)$, $\Upsilon _3 = -0.01093$, with $\mathbb {C}_{b} = (-0.2133, 0.1915)$. In all curve fittings, the confidence bound is $95\%$.

*SSE* : 0.05051, $R-square: 0.9989$, $Adjusted R-square: 0.9921$, and *RMSE* : 0.2247

- **HOF using**  $$R_{-1}(FeTe_{2})$$

$$\begin{aligned} f(R_{-1}) = \frac{\left( \chi _1\times R_{-1}^3 + \chi _2\times R_{-1}^2 + \chi _3\times R_{-1} + \chi _4 \right) }{\left( R_{-1}^3 + \Upsilon _1\times R_{-1}^2 + \Upsilon _2\times R_{-1}^3\right) } \end{aligned}$$

where $R_{-1}$ is standardized by $\mu =21.75$ and $\sigma =17.32$.The Coefficients:$\chi _1 = -2.418$, with $\mathbb {C}_{b} = (-4.742, -0.09399)$, $\chi _2 = -2.418$, with $\mathbb {C}_{b} = (-6.636, 1.8)$, $\chi _3 = 0.1319$, with $\mathbb {C}_{b} = (-1.592, 1.856)$,$\chi _4 = 0.1728$, with $\mathbb {C}_{b} = (-1.749, 2.095)$, $\Upsilon _1 = 1.26$, with $\mathbb {C}_{b} = (0.6277, 1.892)$, $\Upsilon _2 = 0.2355$, with $\mathbb {C}_{b} = (-0.2099, 0.681)$. $\Upsilon _3 = -0.02212$, with $\mathbb {C}_{b} = (-0.2473, 0.2031)$.

*SSE* : 0.04895, $R-square: 0.9989$, $Adjusted R-square: 0.9924$, and *RMSE* : 0.2212.

- **HOF using**  $${R_{\frac{1}{2}}(FeTe_{2})}$$

$$\begin{aligned} f({R_\frac{1}{2})} = \frac{\left( \chi _1\times R_\frac{1}{2}^3 + \chi _2\times R_\frac{1}{2}^2 + \chi _3\times R_\frac{1}{2} + \chi _4\right) }{\left( R_\frac{1}{2}^3 + \Upsilon _1\times R_\frac{1}{2}^2 + \Upsilon _2\times R_\frac{1}{2} + \Upsilon _3\right) } \end{aligned}$$

In which $R_\frac{1}{2}$ is standardized by $\mu =442.7$ and $\sigma =398$. The Coefficients: $\chi _1 = -2.394$, with $\mathbb {C}_{b} = (-4.797, 0.009614)$, $\chi _2 = -2.536$, with $\mathbb {C}_{b} = (-6.767, 1.696)$, $\chi _3 = -0.03775$, with $\mathbb {C}_{b} = (-1.776, 1.701)$, $\chi _4= 0.1773$, with $\mathbb {C}_{b} = (-1.651, 2.006)$, $\Upsilon _1 = 1.31$, with $\mathbb {C}_{b} = (0.7252, 1.896)$, $\Upsilon _2 = 0.3113$, with $\mathbb {C}_{b} = (-0.1102, 0.7327)$, $\Upsilon _3 = -0.01299$, with $\mathbb {C}_{b} = (-0.2189, 0.1929)$.

*SSE* : 0.05024, $R-square: 0.9989$, $Adjusted R-square: 0.9922$, and *RMSE* : 0.2241.

- **HOF using** $$R_{\frac{-1}{2}}(FeTe_{2})$$

$$\begin{aligned} f({R_\frac{-1}{2})} = \frac{\left( \chi _1\times R_\frac{-1}{2}^3 + \chi _2\times R_\frac{-1}{2}^2 + \chi _3\times R_\frac{-1}{2} + \chi _4\right) }{\left( R_\frac{-1}{2}^3 + \Upsilon _1\times R_\frac{-1}{2}^2 + \Upsilon _2\times R_\frac{-1}{2} + \Upsilon _3\right) } \end{aligned}$$

In which $R_\frac{-1}{2}$ is standardized by $\mu =57.48$ and $\sigma =48.42$. The Coefficients: $\chi _1 = -2.408$, with $\mathbb {C}_{b} = (-4.764, -0.052)$, $\chi _2 = -2.467$, with $\mathbb {C}_{b} = (-6.691, 1.757)$, $\chi _3 = 0.06116$, with $\mathbb {C}_{b} = (-1.668, 1.791)$, $\chi _4 = 0.1754$, with $\mathbb {C}_{b} = (-1.707, 2.058)$, $\Upsilon _1 = 1.281$ , with $\mathbb {C}_{b} = (0.6679, 1.894)$, $\Upsilon _2 = 0.2671$ , with $\mathbb {C}_{b} = (-0.169, 0.7032)$, $\Upsilon _3 = -0.01858$ , with $\mathbb {C}_{b} = (-0.2354, 0.1982)$.

*SSE* : 0.04947, $R-square: 0.9989$, $Adjusted R-square: 0.9923$, and *RMSE* : 0.2224.

The Randi$\acute{c}$ index for a specific molecule corresponds to its value, and the coefficients control how the Randi$\acute{c}$ index and the heat of formation are related.

- **HOF using**  $$M_{1} (FeTe_{2})$$

$$\begin{aligned} f(M_{1}) = \frac{\left( \chi _1\times M_{1}^3 + \chi _2\times M_{1}^2 + \chi _3\times M_{1} + \chi _4\right) }{\left( M_{1}^3 + \Upsilon _1\times M_{1}^2 + \Upsilon _2\times M_{1} + \Upsilon _3\right) } \end{aligned}$$

where $M_1$ is standardized by $\mu =891$ and $\sigma =798.7$. The Coefficients: $\chi _1$ = -2.394, with $\mathbb {C}_{b}$ = (-4.796, 0.007124), $\chi _2$ = -2.533 , with $\mathbb {C}_{b}$ = (-6.764, 1.698), $\chi _3$ = -0.03386 , with $\mathbb {C}_{b}$ = (-1.772, 1.704), $\chi _4$= 0.1773, with $\mathbb {C}_{b}$ = (-1.653, 2.008), $\Upsilon _1$ = 1.309, with $\mathbb {C}_{b}$ = (0.7229, 1.896), $\Upsilon _2$ = 0.3095, with $\mathbb {C}_{b}$ = (-0.1125, 0.7316), $\Upsilon _3$ = -0.01322, with $\mathbb {C}_{b}$ = (-0.2195, 0.1931).

*SSE* : 0.0502, $$R-square: 0.9989$$, $$ Adjusted R-square: 0.9922$$, and *RMSE* : 0.2241.

- **HOF using**  $$M_{2}(FeTe_{2})$$

$$\begin{aligned} f(M_{2}) = \frac{\left( \chi _1 \times M_{2}^3 + \chi _2 \times M_{2}^2 + \chi _3 \times M_{2} + \chi _4\right) }{\left( M_{2}^3 + \Upsilon _1\times M_{2}^2 + \Upsilon _2\times M_{2} + \Upsilon _3\right) } \end{aligned}$$

where $M_2$ is standardized by $\mu = 1267$ and $\sigma = 1160$. The Coefficients: $\chi _1$ = -2.389, with $\mathbb {C}_{b}$ = (− 4.81, 0.03131), $\chi _2$ = − 2.559, with $\mathbb {C}_{b}$ = (− 6.793, 1.674), $\chi _3$ = − 0.07126, with $\mathbb {C}_{b}$ = (− 1.813, 1.671), $\chi _4$= 0.1775, with $\mathbb {C}_{b}$ = (− 1.633, 1.988), $\Upsilon _1$ = 1.322, with $\mathbb {C}_{b}$ = (0.7449, 1.896), $\Upsilon _2$ = 0.3262, with $\mathbb {C}_{b}$ = (− 0.08985, 0.7423), $\Upsilon _3$ = − 0.01093, with $\mathbb {C}_{b}$ = (− 0.2133, 0.1915).

*SSE* : 0.05051, $R-square: 0.9989$, $Adjusted R-square: 0.9921$, and *RMSE* : 0.2247.

The objective is to create a mathematical formula or model that can accurately predict the heat of formation using the Zagreb index.

- **HOF using**  $$ABC(FeTe_{2})$$

$$\begin{aligned} f(ABC) = \frac{\left( \chi _1\times ABC^3 + \chi _2\times ABC^2 + \chi _3\times ABC + \chi _4\right) }{\left( ABC^3 + \Upsilon _1\times ABC^2 + \Upsilon _2\times ABC + \Upsilon _3\right) } \end{aligned}$$

In which *ABC* is standardized by $\mu = 112.7$ and $\sigma = 94.31$. The Coefficients: $\chi _1 = -2.278$, with $\mathbb {C}_{b} = (-4.245, -0.3114)$, $\chi _2 = -1.632$, with $\mathbb {C}_{b} = (-5.356, 2.092)$, $\chi _3 = 0.7934$, with $\mathbb {C}_{b} = (-1.128, 2.715)$, $\chi _4= 0.09387$, with $\mathbb {C}_{b} = (-1.229, 1.417)$, $\Upsilon _1 = 0.9576$, with $\mathbb {C}_{b}= (0.2465, 1.669)$, $\Upsilon _2 = -0.2132$, with $\mathbb {C}_{b}= (-0.5772, 0.1507)$, $\Upsilon _3 = -0.1344$, with $\mathbb {C}_{b}= (-0.3936, 0.1248)$.

*SSE* : 0.07398, $R-square: 0.9984$, $Adjusted R-square: 0.9885$, and *RMSE* : 0.2720.

The potential correlation or relationship between the atom bond connectivity index and the heat of formation is investigated by using a curve fitting model.

- **HOF using**  $$GA(FeTe_{2})$$

$$\begin{aligned} f(GA) = \frac{\left( \chi _1\times GA^3 + \chi _2\times GA^2 + \chi _3\times GA + \chi _4\right) }{\left( GA^3 + \Upsilon _1\times GA^2 + \Upsilon _2\times GA + \Upsilon _3\right) } \end{aligned}$$

where *GA* is standardized by $\mu = 156.2$ and $\sigma = 137.3$.

The Coefficients: $\chi _1$ = − 2.399 with $\mathbb {C}_{b}$ = (− 4.784, − 0.01472), $\chi _2$ = − 2.509, with $\mathbb {C}_{b}$ = (− 6.737, 1.719), $\chi _3$ = 0.000643, with $\mathbb {C}_{b}$ = (− 1.734, 1.735), $\chi _4$= 0.1768, with $\mathbb {C}_{b}$ = (− 1.673, 2.026), $\Upsilon _1$ = 1.299, with $\mathbb {C}_{b}$ = (0.7028, 1.895), $\Upsilon _2$ = 0.2941, with $\mathbb {C}_{b}$ = (− 0.1332, 0.7215), $\Upsilon _3$ = − 0.01524, with $\mathbb {C}_{b}$ = (− 0.2253, 0.19948).

*SSE* : 0.04993, $R-square: 0.9989$, $Adjusted R-square: 0.9922$, and *RMSE* : 0.2235.

The heat of formation for new values of the geometric arithmetic index that lie within the range of the data used to fit the curve can be predicted using the curve fitting model once its parameters have been established.

- **HOF using** $$PM_{1}(FeTe_{2})$$

$$\begin{aligned} f(PM_1) = \frac{\left( \chi _1\times PM_1^4 + \chi _2\times PM_1^3 + \chi _3\times PM_1^2 + \chi _4\times PM_1 + \chi _5\right) }{\left( PM_1 + \Upsilon _1\right) } \end{aligned}$$

In which $PM_1$ is standardized by $\mu = 1.334e+09$ and $\sigma = 1.913e+09$.

The Coefficients: $\chi _1$ = 36.73, with $\mathbb {C}_{b}$ = (− 572.5, 646), $\chi _2$ = − 65.91, with $\mathbb {C}_{b}$ = (− 1156, 1024), $\chi _3$ = − 31.2, with $\mathbb {C}_{b}$ = (− 551.4, 489), $\chi _4$ = 27.52, with $\mathbb {C}_{b}$ = (− 457.4, 512.5), $\chi _5$ = 1.853, with $\mathbb {C}_{b}$ =(− 44.13, 47.84), $\Upsilon _1$ = 1.565, with $\mathbb {C}_{b}$ = (− 16.02, 19.15).

*SSE* : 0.4302, $R-square: 0.9904$, $Adjusted R-square: 0.9425$, and *RMSE* : 0.6559.

- **HOF using**  $$PM_{2}(FeTe_{2})$$

$$\begin{aligned} f(PM_{2}) = \frac{\left( \chi _1\times PM_{2}^4 + \chi _2\times PM_{2}^3 + \chi _3\times PM_{2}^2 + \chi _4\times PM_{2} + \chi _5\right) }{\left( PM_{2} + \Upsilon _1\right) } \end{aligned}$$

where $PM_2$ is standardized by $\mu = 1.2e+09$ and $\sigma = 1.721e+09$.

The Coefficients: $\chi _1$ =$-2.216e+05$, with $\mathbb {C}_{b}$ =$(-3.415e+10, 3.415e+10)$, $\chi _2$ = $3.967e+05$, with $\mathbb {C}_{b}$ = $(-6.114e+10, 6.114e+10)$, $\chi _3$ =$1.883e+05$, with $\mathbb {C}_{b}$ = $(-2.902e+10, 2.902e+10)$, $\chi _4$ =$-1.767e+05$, with $\mathbb {C}_{b}$ = $(-2.724e+10, 2.724e+10)$, $\chi _5$ =$-1.554e+04$, with $\mathbb {C}_{b}$ = $(-2.394e+09, 2.394e+09)$, $\Upsilon _1$= $-6498$, with $\mathbb {C}_{b}$ = $(-1.002e+09, 1.002e+09)$.

*SSE* : 0.6218, $R-square: 0.9861$, $Adjusted R-square: 0.9169$, and *RMSE* : 0.7886.

By applying a curve fitting model, it is possible to produce a mathematical representation that can help understand and forecast the relationship between the various Zagreb indices and the heat of formation. The features of molecule structure that influence energy stability may become clearer as a result.

- **HOF using**  $$HM(FeTe_{2})$$

$$\begin{aligned} f(HM) = \frac{\left( \chi _1 \times HM^3 + \chi _2\times HM^2 + \chi _3\times HM + \chi _4\right) }{\left( HM^3 + \Upsilon _1\times HM^2 + \Upsilon _2\times HM + \Upsilon _3\right) } \end{aligned}$$

In which *HM* is standardized by $\mu = 5113$ and $\sigma = 4663$.

The Coefficients: $\chi _1$ = -2.39, with $\mathbb {C}_{b}$ = (-4.808, 0.02739), $\chi _2$ = -2.555, with $\mathbb {C}_{b}$ = (-6.788, 1.678), $\chi _3$ = -0.06526, with $\mathbb {C}_{b}$ = (-1.807, 1.676), $\chi _4$= 0.1775, with $\mathbb {C}_{b}$ = (-1.636, 1.991), $\Upsilon _1$ = 1.319, with $\mathbb {C}_{b}$ = (0.7414, 1.896), $\Upsilon _2$ = 0.3235, with $\mathbb {C}_{b}$ = (-0.09351, 0.7406), $\Upsilon _3$ = -0.0113, with $\mathbb {C}_{b}$ = (-0.2143, 0.1917).

*SSE* : 0.05046, $R-square: 0.9989$, $Adjusted R-square: 0.9921$, and *RMSE* : 0.2246.

- **HOF using**  $$F(FeTe_{2})$$

$$\begin{aligned} f{(F)} = \frac{\left( \chi _1\times F^3 + \chi _2\times F^2 + \chi _3\times F + \chi _4\right) }{\left( F^3 + \Upsilon _1\times F^2 + \Upsilon _2\times F + \Upsilon _3\right) } \end{aligned}$$

where *F* is standardized by $\mu = 2580$ and $\sigma = 2343$.

The Coefficients: $\chi _1$ = $-2.391$, with $\mathbb {C}_{b}$ = $(-4.805, 0.02353)$, $\chi _2$ = $-2.551$, with $\mathbb {C}_{b}$ = $(-6.783, 1.682)$, $\chi _3$ = $-0.05932$, with $\mathbb {C}_{b}$ = $(-1.8, 1.681)$, $\chi _4$= 0.1775, with $\mathbb {C}_{b}$ = $(-1.639, 1.994)$, $\Upsilon _1$ = 1.317, with $\mathbb {C}_{b}$ = (0.7379, 1.896), $\Upsilon _2$ = 0.3209, with $\mathbb {C}_{b}$ = $(-0.09713, 0.7389)$, $\Upsilon _3$ = $-0.01167$, with $\mathbb {C}_{b}$ = $(-0.2153, 0.1919)$.

*SSE* : 0.05041, $R-square: 0.9989$, $Adjusted R-square: 0.9921$, and *RMSE* : 0.2245.

A curve fitting model is used to establish a relationship between the forgetting index and heat of creation. This investigation improves knowledge of the molecule’s energy properties.

- **HOF using**  $$J(FeTe_{2})$$

$$\begin{aligned} f{(J)} = \frac{\left( \chi _1\times J^3 + \chi _2\times J^2 + \chi _3\times J + \chi _4\right) }{\left( J^3 + \Upsilon _1\times J^2 + \Upsilon _2\times J + \Upsilon _3\right) } \end{aligned}$$

where *J* is standardized by $\mu = 213.4$ and $\sigma = 162.1$.

The Coefficients: $\chi _1 = -2.427$, with $\mathbb {C}_{b}=(-4.716, -0.1388)$, $\chi _2= -2.371$, with $\mathbb {C}_{b}=(-6.566, 1.824)$, $\chi _3= 0.1789$ , with $\mathbb {C}_{b} = (-1.531, 1.889)$, $\chi _4= 0.1658$, with $\mathbb {C}_{b} = (-1.764, 2.096)$, $\Upsilon _1 = 1.241$, with $\mathbb {C}_{b} = (0.5953, 1.888)$, $\Upsilon _2 = 0.214$, with $\mathbb {C}_{b} = (-0.236, 0.6641)$, $\Upsilon _3 = -0.02336$, with $\mathbb {C}_{b} = (-0.2519, 0.2052)$.

*SSE* : 0.04838, $R-square: 0.9989$, $Adjusted R-square: 0.9925$, and *RMSE* : 0.22.

The topological complexity or shape of a molecule is measured by the Balaban index, a molecular graph descriptor. The heat of formation is a measurement of the energy change that takes place during the production of a compound.

- **HOF using** $$AZI(FeTe_{2})$$

$$\begin{aligned} f{(AZI)} = \frac{\left( \chi _1\times AZI^3 + \chi _2\times AZI^2 + \chi _3\times AZI + \chi _4\right) }{\left( AZI^3 + \Upsilon _1\times AZI^2 + \Upsilon _2\times AZI + \Upsilon _3\right) } \end{aligned}$$

where *AZI* is standardized by $\mu = 1641$ and $\sigma = 1487$.

The Coefficients: $\chi _1$ = $-2.391$, with $\mathbb {C}_{b}$ = $(-4.804, 0.02164)$, $\chi _2$ = $-2.549$, with $\mathbb {C}_{b}$ = $(-6.781, 1.684)$, $\chi _3$ = $-0.0564$, with $\mathbb {C}_{b}$ = $(-1.797, 1.684)$, $\chi _4$ = 0.1775, with $\mathbb {C}_{b}$ = $(-1.641, 1.996)$, $\Upsilon _1$ = 1.316 , with $\mathbb {C}_{b}$ = (0.7362, 1.896), $\Upsilon _2$ = 0.3196 , with $\mathbb {C}_{b}$ = $(-0.0989, 0.7381)$, $\Upsilon _3$ = $-0.01185$ , with $\mathbb {C}_{b}$ = $(-0.2158, 0.1921)$.

*SSE* : 0.05039, $R-square: 0.9989$, $Adjusted R-square: 0.9921$, and *RMSE* : 0.2245.

The steps in the curve fitting process include selecting an appropriate model, calculating its parameters using the available data, and assessing the model’s goodness of fit. Thus, the fitted curve may be used to forecast the link between the improved Zagreb index and heat of formation and can also be used for analysis.

## Discussion

In this work, we primarily characterised a number of topological indices for an Iron Telluride network, including as the Balaban index, the Forgotten index, the Zagreb type indices, and the generic Randic index. Chemical graph theory makes extensive use of these indices to measure molecular structural properties by looking at patterns of connectedness. Our objective was to have a better understanding of the structural characteristics and behaviour of the Iron Telluride network through the statistical study of these variables.

The universal Randic index is a topological metric that sums the reciprocal square roots of the bond degrees of each vertex in the molecular graph to describe the connectivity and branching of a molecule. In contrast, the Zagreb type indices measure the sum of the squares of the degrees of every vertex in a molecular network. They are based on the vertex degree. These indices have been utilised in QSAR/QSPR investigations to predict the biological and physicochemical features of compounds. By adding up the products of the bond degrees of each pair of vertices in the molecular graph, the Balaban index is a topological metric that assesses the branching and chemical complexity of a molecule. It is a measurement of a molecule’s topological symmetry and has been used to predict different characteristics of compounds in QSAR/QSPR investigations.

We computed these topological indices for the Iron Telluride network in our study and performed statistical analysis to comprehend their correlations and importance in forecasting the network’s chemical and structural characteristics. We examined the connections between these indices and other characteristics of the Iron Telluride network, such as its Gibbs energy and thermodynamic features, using curve fitting techniques like linear regression.

## Conclusion

Our study’s findings on the characterization of the Iron Telluride network’s topological indices and statistical analysis using curve fitting models offer insightful information that might be used for the creation of new materials. The vertex partition method, edge partition method, graph theoretical tools, analytic approaches, degree counting method, sum of degrees of neighbors method, and combinatorial computing method are the methods we employ to compute our results for $FeTe_2$. In addition, we plot graphical representation of these mathematical results using Maple and do mathematical computations using MATLAB. Investigating topological indices offers a thorough comprehension of the network’s connectivity patterns, illuminating its distinct characteristics and possible uses. The statistical analyses that are carried out provide a quantitative viewpoint on the observed properties and lay the groundwork for further research, which adds to the larger scientific conversation. This article’s results not only improve our understanding of the Iron Telluride network but also highlight the importance of topological indices in materials science investigations. The methods and insights presented here open up new avenues for advancements in the field as we continue to decipher the molecular details of various materials.

## Data Availability

The datasets used and/or analysed during the current study available from the corresponding author on reasonable request.
